# Using mixed methods to construct and analyze a participatory agent-based model of a complex Zimbabwean agro-pastoral system

**DOI:** 10.1371/journal.pone.0237638

**Published:** 2020-08-21

**Authors:** M. V. Eitzel, Jon Solera, K. B. Wilson, Kleber Neves, Aaron C. Fisher, André Veski, Oluwasola E. Omoju, Abraham Mawere Ndlovu, Emmanuel Mhike Hove

**Affiliations:** 1 Science and Justice Research Center, University of California, Santa Cruz, Santa Cruz, CA, United States of America; 2 Seven Points Consulting, Lafayette, CA, United States of America; 3 The Muonde Trust, Mazvihwa Communal Area, Midlands Province, Zimbabwe; 4 Universidade Federal do Rio de Janeiro, Rio de Janeiro, Brazil; 5 Lawrence Livermore National Laboratory, Livermore, CA, United States of America; 6 Tallinn University of Technology, Tallinn, Estonia; 7 National Institute for Legislative and Democratic Studies (National Assembly), Maitama, Abuja, Nigeria; Michigan State University, UNITED STATES

## Abstract

Complex social-ecological systems can be difficult to study and manage. Simulation models can facilitate exploration of system behavior under novel conditions, and participatory modeling can involve stakeholders in developing appropriate management processes. Participatory modeling already typically involves qualitative structural validation of models with stakeholders, but with increased data and more sophisticated models, quantitative behavioral validation may be possible as well. In this study, we created a novel agent-based-model applied to a specific context: Zimbabwean non-governmental organization the Muonde Trust has been collecting data on their agro-pastoral system for the last 35 years and had concerns about land-use planning and the effectiveness of management interventions in the face of climate change. We collaboratively created an agent-based model of their system using their data archive, qualitatively calibrating it to the observed behavior of the real system without tuning any parameters to match specific quantitative outputs. We then behaviorally validated the model using quantitative community-based data and conducted a sensitivity analysis to determine the relative impact of underlying parameter assumptions, Indigenous management interventions, and different rainfall variation scenarios. We found that our process resulted in a model which was successfully structurally validated and sufficiently realistic to be useful for Muonde researchers as a discussion tool. The model was inconsistently behaviorally validated, however, with some model variables matching field data better than others. We observed increased model system instability due to increasing variability in underlying drivers (rainfall), and also due to management interventions that broke feedbacks between the components of the system. Interventions that smoothed year-to-year variation rather than exaggerating it tended to improve sustainability. The Muonde trust has used the model to successfully advocate to local leaders for changes in land-use planning policy that will increase the sustainability of their system.

## Introduction

Studying and managing complex social-ecological systems is difficult because they can behave unpredictably, exhibiting time lags between components, sudden shifts between alternative stable states, and nonlinearity in response to system drivers, among other challenges. [1] Adaptive management of these systems constitutes a series of observational studies with potentially uncertain results—and yet the stakes are typically high and action is required, a situation calling for a “post-normal” research approach that can suggest or support actions even when uncertainty cannot be eliminated. [2] Simulation studies are a common strategy for addressing these challenges, facilitating exploration of complex system responses to current conditions as well as their possible responses to changing system drivers in the future. [3, 4] Even with these tools, complex social-ecological systems can also be notoriously “wicked” to manage, in the sense that defining the problem to be solved can be elusive and vary from stakeholder to stakeholder, attempted solutions may cause as many problems as they solve, and each attempt has consequences that cannot be ignored. [5] Collaborative research strategies can be key in working with these challenges. [4]

Bringing together simulation modeling strategies with community collaboration has been referred to by many names, but can be broadly termed “participatory modeling.” [6] These processes can involve focus groups, role-playing games, workshops, and many other types of community engagement, and can involve community members in some or all parts of the modeling process (including goal setting, data collection, design, implementation, verification and calibration, validation, use, and outcome analysis). [7] Many participatory modeling methods focus on the questions and needs of the community the model is meant to serve, and perhaps the most important criterion for a participatory simulation’s validity is its credibility to and/or usefulness for its users, often assessed through an interactive, relational process. [8–10] However, as communities themselves are becoming more sophisticated in their collection and use of data to address questions that concern them, [11] quantitative methods of external validation may become desirable in addition to more internal methods associated with usefulness. Communities may want to see model results match their quantified experience of their system, in addition to the qualitative assessment of usefulness and credibility. There may be a corresponding need for more quantified validation and analysis techniques, often seen in other modeling domains (for example, systems dynamics modeling [12]).

Many of the models created by these collaborative approaches are agent-based simulations (or agent-based models, ABMs) in which the behavior of individual entities is modeled, typically including the entities’ interactions with a spatially-explicit landscape. [13] Unfortunately ABMs are often criticized for being difficult to validate. [14] Ideally, the process of ensuring that a simulation model is ‘good enough’ should include ‘verification’ steps (checking that the model behaves as expected) and ‘validation’ steps (checking whether the model is a reasonable representation of the system it is meant to echo). [15] Typically, verification of ABMs can be a combination of ‘face validation,’ a process of watching model runs, tracking individual agents or landscape patches, and a coarse assessment of whether the outputs fall within a reasonable range, [16] and other more granular processes like the software best practice of ‘unit tests’ that verify individual functions of the model through testing known inputs against expected outputs. [15] Other stages of a typical model assessment process include calibration of model parameters, output validation against independent field data, and sensitivity analysis to identify parameters with disproportionate impact on model outcomes. [16] System dynamics modelers have distinguished two stages of validation: a largely qualitative “structural” phase in which users ensure that the model includes all the important entities and processes with appropriate causal mechanisms, and a “behavioral” phase which is more quantitatively compared with the performance of the real system being modeled. [17] Participatory modeling often engages with structural validation processes, but as quantitative data are increasingly available via processes like citizen science, [11] behavioral validation is becoming more feasible as well.

In this study, we engaged in a participatory modeling process with Zimbabwean non-governmental organization The Muonde Trust to address their concerns around land-use decisions and management practices in their agro-pastoral system. Our model was designed to support a discussion process between Muonde farmer-researchers and other community members as well as local leaders in Mazvihwa Communal Area. There are several examples of excellent agent-based models of Zimbabwean social-ecological system management, but they are only structurally validated. [18, 19] Because Muonde’s research team has accumulated a relatively large amount of quantitative data for this type of sub-Saharan African system, we sought to both structurally and behaviorally validate our model. Below, we describe the model and the results of this validation process: the model was successfully structurally validated by the community, and quantitative behavioral validation was more successful for some model targets than others.

## Materials and methods

Below, we briefly describe the study system and community-based research team, our collaborative modeling process, and the model itself. We then discuss model assessment, and finally describe our graphical and statistical methods for analyzing model results. We note that this project was conducted in the context of decolonial research, intending to support local collective governance of a social-ecological system, but the focus of this paper is on our model construction and validation process.

### Study system: Mazvihwa Communal Area, Zimbabwe and the Muonde Trust

This study is based in Mazvihwa Communal Area, Midlands Province, south-central Zimbabwe. The ecosystem in Mazvihwa is semi-arid, with highly variable within-year and between-year rainfall. The land is classified in the lowest-potential agricultural zone of the country, and farmer-pastoralists living in the Communal Area have survived using a variety of strategies to manage livestock, crops, and woodland grazing area, including storing grain, subsidizing livestock, and moving livestock to better forage locations on multiple temporal and spatial scales. In addition, the Muonde Trust, a local non-governmental organization composed of members from several villages around Mazvihwa, has been developing and promoting new innovations, including cultivation of Indigenous small grains, water harvesting techniques, dry stone-walling, and re-foresting the woodland grazing areas. Muonde’s research team includes individuals from a range of clans and backgrounds, with more women members than men. Muonde seeks to answer questions regarding the consequences of both existing management techniques as well as the newer innovations on the sustainability of their agro-ecosystem.

### Collaborative modeling process

Following [7], we break down the community’s involvement in each stage of the modeling process, assessing each stage as ‘community-driven,’ ‘collaborative,’ or ‘outsider-driven.’ The Muonde Trust research team has been recording data on a variety of aspects of their agro-pastoral system over multiple decades. Both Muonde founders (A. Mawere Ndlovu and K.B. Wilson) have regularly interviewed farmers in Mazvihwa over the last 35 years, recording practices and observations about system behaviors. They and other Muonde researchers (E. Mhike Hove, among others) have also quantitatively measured a variety of aspects of the agro-pastoral system (*community-driven data collection*). Wilson has kept an archive of these data, representing a rich collection of quantitative and qualitative expert knowledge of the system. [20] Driven by the community’s recent concerns about dwindling woodland grazing area as more and more land is converted to arable crop production (*community-driven diagnosis/synthesis and determination of modeling goals*), Muonde engaged with quantitative modelers (M.V. Eitzel) to explore what kinds of answers could be found using the Muonde data archive.

Eitzel, Wilson, and Mawere Ndlovu chose an Agent-Based Modeling framework to be used as a discussion tool for Muonde and local communities in Mazvihwa, and conceptualized a representation of the important entities and feedbacks in the Mazvhiwa agro-pastoral system (*collaborative conceptual model design*). Eitzel, with the help of other modelers (K. Neves, O. Omoju, A. Veski), implemented the model in NetLogo [21] as part of the Santa Fe Institute’s Complex Systems Summer School (*outsider-driven implementation*). Calibration via including additional model behaviors and refining specific model parameters was an iterative process between Wilson, Mawere Ndlovu, and Eitzel (*collaborative calibration*). The model was then presented to the larger Muonde research team (around 30 people) in several workshops. These included whole-group discussions of the Mazvihwa agro-pastoral system, followed by a series of small-group (5-10 people) hands-on experiences of running and discussing the model, concluding with a whole-group discussion of the model. Between the calibration and verification with Wilson, Mawere Ndlovu, and Eitzel, and the additional discussion with the Muonde research team, the overall structural validation of the model was collaborative (*collaborative verification and simulation*). The model has been discussed in additional workshops run by the Muonde team for local leaders following a similar format to the earlier workshops, and the results of the model are being analyzed and presented in multiple academic publications (*collaborative discussion of results and community-driven use of the model*). The model was also peer reviewed and archived in the model library of the Network for Computational Modeling in Social and Ecological Sciences (CoMSES.net, [22, 23]).

For the results analyzed in this paper, Eitzel ran a large parameter sweep of the model with the help of high-performance computing experts J. Solera and A.C. Fisher (*outsider-driven verification and simulation process*) and conducted a final quantitative behavioral validation check against Muonde’s field data (*outsider-driven validation*).

### Model description

**Fig 1:**
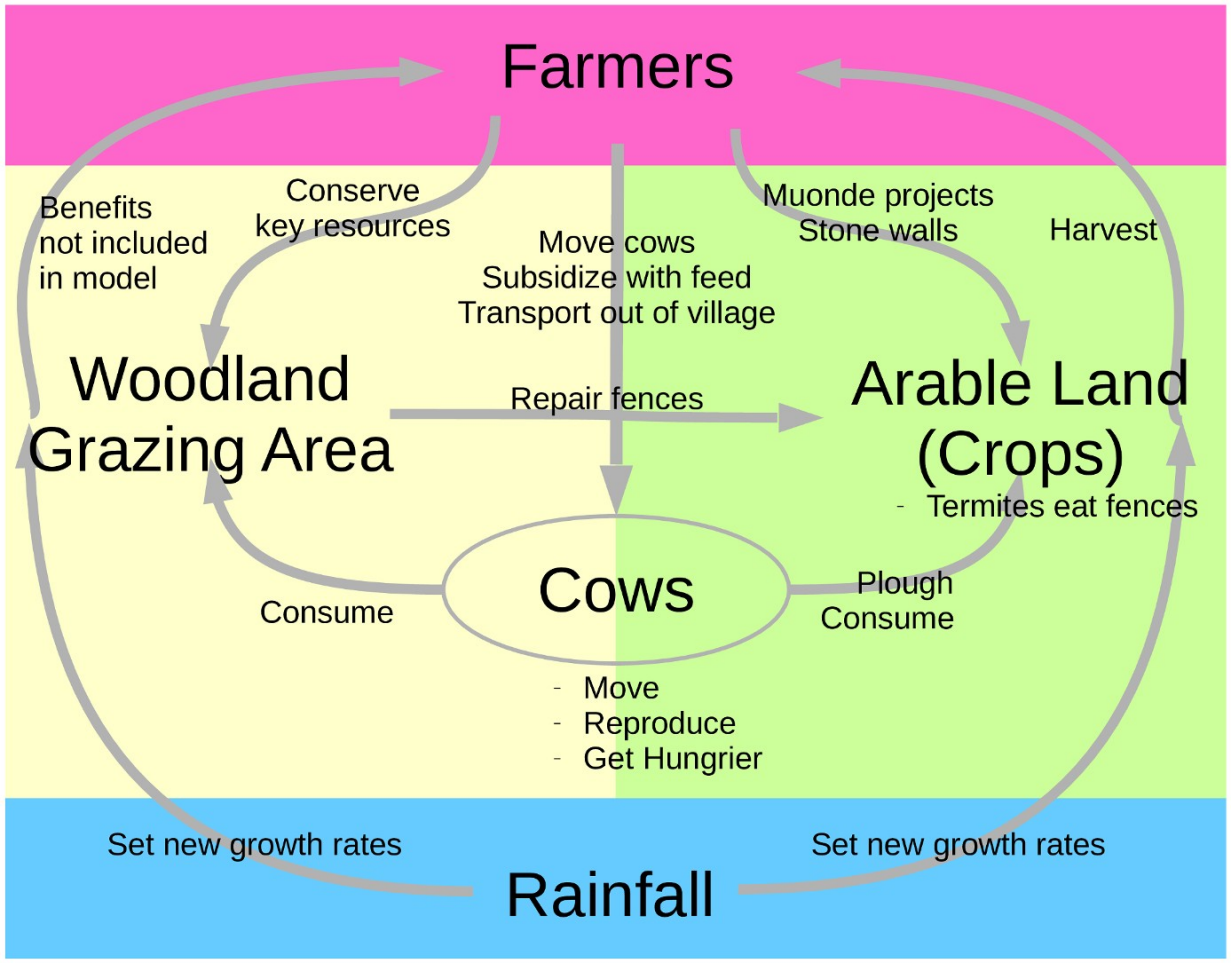
Diagram representing the feedbacks in the model. Farmers (represented by the model user) control a variety of aspects of the system in a top-down fashion, while rainfall determines many model behaviors in a bottom-up fashion by influencing how much biomass is available in the system. Cows are modeled as agents, moving through crop and woodland patches. Crops depend on cows through ploughing, crops depend on woodland for fencing material, cows depend on woodland or crops for food intake, and cows reproduce periodically. (Figure from [23]).

In order to answer the community’s questions about management interventions and land-use planning, we created a model that simulates livestock (cattle, specifically, due to their cultural importance), crop fields, and woodland grazing area. Because the model was intended to be used as a discussion tool, implemented as a kind of computer-mediated role-play for community members and local leaders, farmers are represented in the model by the choices the user makes in the modeling interface. The model’s parameters and behaviors integrate a variety of data sources, including community-sourced quantitative and qualitative data from the Muonde Trust research team, as well as rainfall data from the Zimbabwean government and additional parameters drawn from the literature. (See S1 Appendix for details on data sources and parameter values.) The model incorporates biomass and energy accounting between trophic levels, a two-stage population model for cows, and rainfall-dependent crop and woodland growth. We model several feedbacks between cows, crops, and woodland, including the following: crops depend on cows through ploughing, crops depend on woodland for fencing material, and cows depend on woodland or crops for food intake (Fig 1). We also include many of the traditional and recently-innovated management interventions employed by farmers in Mazvihwa, and simulate several different inter-annual rainfall variation scenarios. See below for more information, and S1 Appendix for details.

To make the model more familiar to users, we chose to base it on a single village in Mazvihwa: Mudhomori, which is estimated to be 600 hectares in size. The model runs for the length of our historical rainfall data time-series (60 calendar years): this allows users who were alive during that time period to recognize historical droughts and allows the model system time to experience multiple droughts and (possibly) recover from them. We chose an 8-hour time step and a 0.24-ha spatial resolution to allow cow movements to be realistic (both in terms of linear distance traveled and amount of field ploughed in a single time step) and to capture the processes of interest [20]—while also making computation feasible.

To ensure energy balance, we account for biomass exchange between primary producers (crops and woodlands) and consumers (cows) by tracking an energy pool for each cow which reflects metabolic and working costs as well as the energy gains from consumption of plant matter. We then use energy densities for each of these types of biomass, and efficiencies in building new tissue or burning reserves when cows aren’t able to eat, in order to convert that energy surplus or deficit to a mass gain or loss. To correctly account for the different weight ranges of adults and juveniles, we adopt a simple two-stage population model for cows (for simplicity, we do not distinguish males from females but give all adult cows a constant probability of reproducing).

We estimate crop growth dependence on rainfall directly from community-based field data and Zimbabwean governmental rainfall records. Though many different crops are grown in Mazvihwa, differentiating between them was not necessary for the model to be useful as a discussion tool regarding coarse land-use choices between arable cultivation and woodland grazing area. For the growth rate of woodland, we have community-based data on growth of acacia and mopane trees, and rely on literature review for herbaceous growth estimates as well as for comparison with our field-based measurements of woody growth. Similar to the crop simplification, we pool all woody biomass regardless of dominant tree species. To arrive at a total woodland biomass growth rate we estimate herbaceous and woody primary production separately and then pool them.

**Fig 2:**
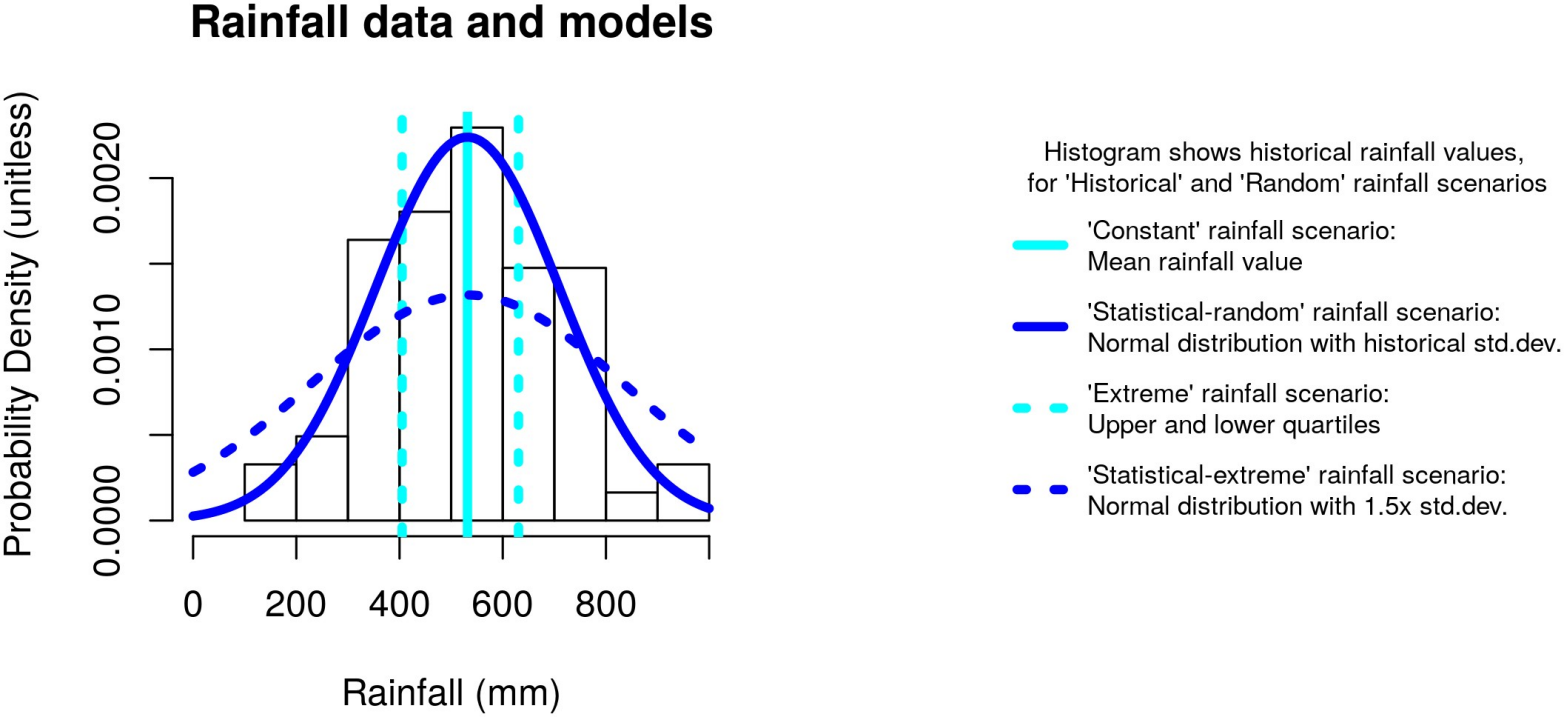
Rainfall data and representations of the different rainfall scenarios. The histogram bars represent the historical rainfall: ‘historical’ and ‘random’ baseline rainfall scenarios use these values (in order, or randomly drawn, respectively). The lines represent the four other rainfall scenarios. (Figure from [23]).

We use historical annual rainfall records to represent realistic water limitations on the system. For validation, we compare independent community field data to outputs of models using these historical rainfall scenarios. However, the Muonde research team is aware of and concerned about the potential impacts of climate change on their system, and climate models do predict increasing rainfall variation for Zimbabwe. [24] Downscaled climate models for southern Africa indicate an increase in rainfall variation around 1.5 times the current standard deviation, [25, 26] so we created two relatively simple ways to simulate increased year-to-year variation with 1.5 times the standard deviation of the historical rainfall data and compared these scenarios to four different ‘baseline’ methods (Fig 2). In reality, within-year variation and extremes such as droughts and erosive events can be much more damaging than year-to-year variation, however our rainfall scenarios were sophisticated enough to generate discussion for the community.

The model user (representing local farmers and leaders) determines the proportion of crops, their spatial configuration, and what percentage of crops or woodland grow faster than the rest (through farming innovations or woodland restoration projects). They determine how many times per day a cow is moved to a better grazing location, whether cows should be subsidized in low-rainfall years (and in what way: by transportation out of the village or by augmentation of their diet with supplemental feed), and what proportion of the cows to subsidize. Farmers also determine whether fences should be ‘invincible’ (meaning stone walls instead of brush fencing) and how long to store crops.

Finally, because the community discussion was around system sustainability, we classified each model run as ‘sustainable’ if all three components (cows, crops, and woodland) met a minimum quantity for all 60 model years of the simulation, rather than focusing on any one of the three components. The logic behind these minimal thresholds is that farmers would not be able to buy cows, seed, or nursery-raised woodland trees, and instead would need at least so many cows, so much seed, or so much woodland biomass for these components to maintain themselves. We recognize that in reality these thresholds would represent an untenably optimistic definition of ‘sustainability,’ but they are adequate for the purpose of exploring model behavior in the validation and sensitivity analysis processes we outline below.

### Model assessment

**Fig 3:**
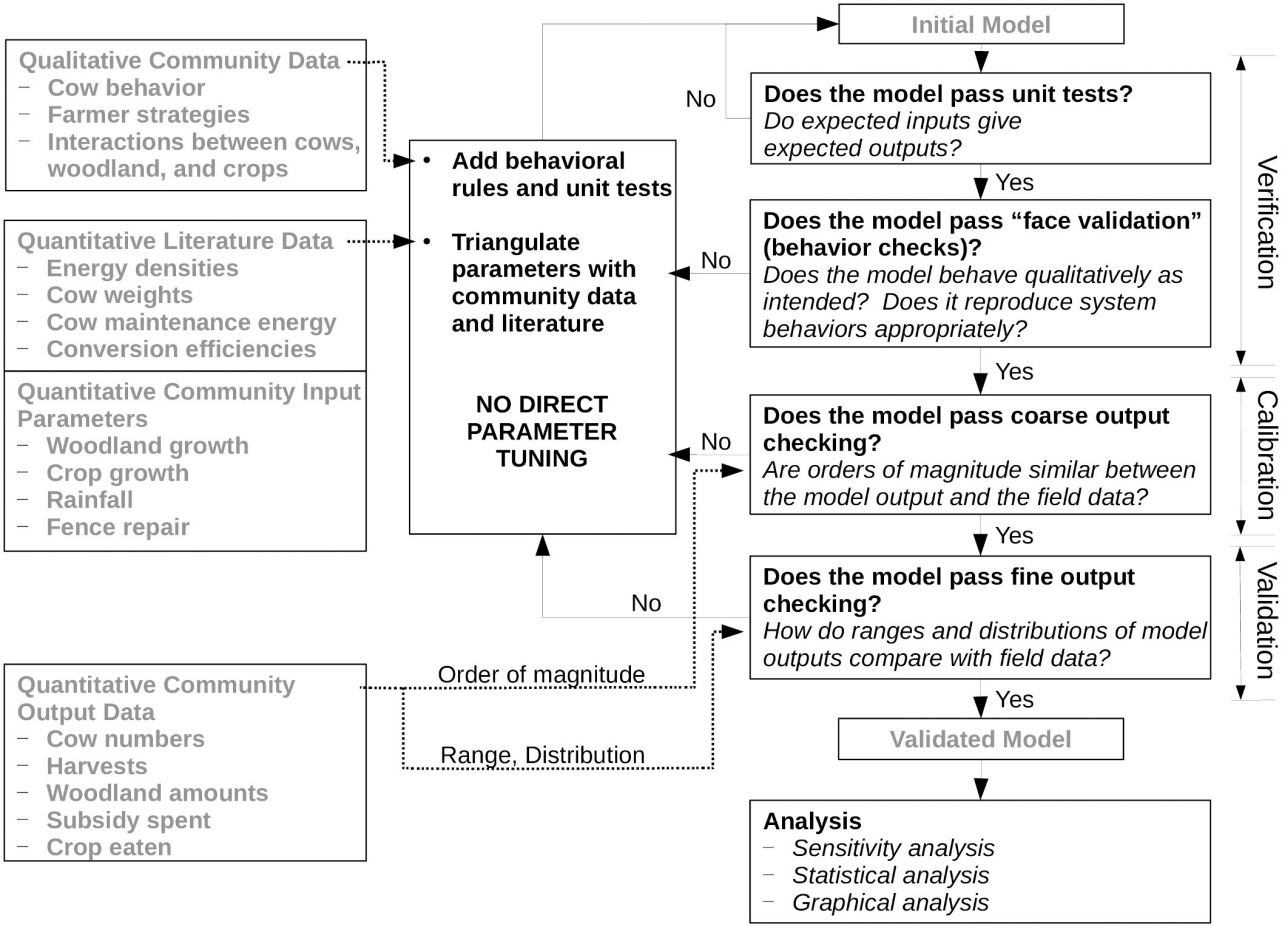
Diagram showing our model construction and validation process. Modeling actions are indicated in bold text; data and models are indicated in light gray text. The flow of modeling steps is indicated with thin solid arrows, showing the iterative nature of our verification, calibration, and validation process, and the entry points for community- and literature-based data are shown with thick dashed arrows. The Muonde research team generated the data labeled ‘Community Data’ and were involved in several iterations of the ‘verification’ stage, while Muonde leaders also worked on the ‘calibration’ stage. Compare with Figure 10.1 in [16]. Note that the ‘verification’ and ‘calibration’ stages constitute structural validation, while the stage labeled ‘validation’ constitutes behavioral or empirical validation.

We assessed our model using both community-based structural validation as well as outsider-driven quantitative behavioral validation, including high-performance computing parameter sweeps and statistical and graphical sensitivity analysis (Fig 3).

#### Qualitative structural validation (verification and calibration)

Model verification included “face validation,” watching model runs (“animation assessment”) and checking individual agents’ and patches’ parameters as they changed dynamically throughout the simulations (“immersive assessment”), coarse order-of-magnitude checks on model outputs (“output assessment”), [16] software unit tests, and comparing model performance against a checklist of expected model behaviors. Through our face validation and unit testing, as well as profiling (checking run times of individual model functions), we reduced the running time of the model to allow for verification parameter sweeps as well as the full sensitivity testing sweeps described below. See S2 Appendix and the ODD on CoMSES.net for additional information on these processes. [23]

To calibrate the model, we began with the simplest version of the system including just the three resources and their interactions with each other, and then we added major management interventions until the coarse behavior of the model matched the coarse behavior of the system (e.g. cow population crashes during droughts, fences being unable to be rebuilt when the woodland was too denuded—using multiple patterns in the real system to guide model design [27]). We then calibrated specific model parameter values based on community-based data and literature. In selecting these values, we have avoided using any tuning parameters to force the model to behave in line with the real system. Instead, we have carefully selected underlying parameters based on literature and field data collected by Muonde’s research team from interviews with farmers, triangulating on model parameters with weaker literature support. We frequently iterated between calibration and verification as we added management interventions and updated parameters with better literature support. [28] These stages of model structural validation resulted in a model that was sufficiently detailed to be useful to the community, but we also wished to explore the potential for behavioral validation as well, so we engaged in an additional process of parameter sweeps, quantitative empirical validation, and sensitivity analysis.

#### Parameter sweeps

To analyze the results of our model, we ran a large parameter sweep with many replications (499,200 runs). This sweep included varying the proportion of land area committed to agriculture (‘proportion-crops’) randomly in each run from 1-99%, perturbing underlying biological parameters by 5% above or below their stated values (as recommended for local sensitivity analysis, [13] with the exception of two variables we perturbed by 10%: first, woodland growth rate, to encompass both the literature and community based data values; and second, how much faster crops with Muonde projects grow, because the Muonde team had not yet measured this value through field experiments), choosing a set of crop ‘clumpiness’ values which gave a relatively even distribution of spatial configurations (measured using Moran’s I [29]), and an equal number of runs in each combination of management interventions and rainfall scenarios. We used NetLogo’s BehaviorSpace functionality to generate these runs, and ran 100 replications of each combination of rainfall scenarios and management interventions, in order to average across random spatial configurations.

To run these parameter combinations and replications, we used Python scripts on a commercial computer cluster, Sabalcore, to launch multiple headless instances of NetLogo’s BehaviorSpace module using qsub (Sabalcore’s queuing system). We used 10,218 core-hours on a set of nodes with 24 2.7 GHz Dual Intel Xeon cores each and 125 GB of RAM per node. Sample code can be found on GitHub. [23]

#### Quantitative behavioral validation

To quantitatively validate our model, we selected model runs which most closely matched the historical system behavior: those that used the ‘historical’ rainfall scenario, met the criteria of finishing a run (lasting all 60 model years), and had realistic management parameters (see S1 Appendix for the specific values). Our field data were not sufficiently complete to conduct direct comparisons of model and real time-series, therefore we sought relatively simple indicators with which to measure differences. For yearly harvest and cow numbers, we could compare the distributions of model data with field data, so we used the method of percent difference in mean and standard deviation. [17, 30] We also checked the order of magnitude of three other quantities for which we could calculate a single number from our field data: the minimum amount of woodland biomass, the amount of money spent on subsidizing cows, and the amount of crop eaten by animals that break into crop fields (see S1 Appendix for the details of these calculations). We compared these with the distribution of similar quantities from each model run; these comparisons are shown in S3 Appendix.

#### Graphical and statistical analysis of parameter sweeps

In our statistical sensitivity analysis, we tested for the relative magnitudes of the impact of the underlying variables at the same time as we tested for the impact of rainfall scenarios and management interventions. We note that the use of sample-size-based statistical tests on simulation results is not particularly meaningful given that the number of runs can always be increased, leading to lower standard errors and higher significance levels. Therefore we focus mainly on parameter estimates, using a Generalized Additive Model (GAM) as a summary tool to explore the large volume of model results. We fit a binomial GAM, using the “mgcv” package (version 1.8-23) [31] in R (version 3.4.4), [32] for whether the cows, crops, and woodland all met the minimum criteria for all 60 model years (a measure of the sustainability of the system), in order to see which variables were statistically significant and what their relative importance was (via the relative magnitudes of their statistical parameter estimates).

For the underlying variables (those from data or literature, e.g. growth rate of woodlands, energy density of browse, and so on) that we had perturbed by ±5-10%, we used a local linear approximation (appropriate for small perturbations). To test the importance of proportion-crops and Moran’s I in the GAM, we used smooth functions (splines) because we examined a wide range of these variables and a local linear approximation was not appropriate. We chose Moran’s I (among the four spatial variables we calculated; see S1 Appendix) for the statistical analysis because it is a classic landscape ecology indicator used to represent spatial diversity, and was least correlated with the proportion-crops. We do graphically analyze the other spatial configuration variables; see below. We also scaled each of the continuous predictor variables to enhance comparability of parameter estimates, as well as centering them to improve the interpretability of the base case (what R estimates as the model’s intercept; see below). Finally, for the discrete management variables and rainfall scenarios, we used categorical factors (e.g. farmers move cows = ‘yes/no’). In our results, because each simulation’s sustainability is a binary variable (0 or 1), we report untransformed parameter estimates in order to compare the magnitude of different model parameters’ influence on model results, but we also discuss transformed parameters on the probability scale to get a better sense of the impact of that parameter.

Our graphical analysis also examined the effects of the variables on the modeled system’s sustainability, analyzing rainfall scenarios separately. Within each rainfall scenario, we chose one of the continuous variables, either the proportion of crops or one of the landscape configuration variables, and then binned the models into 10 equal divisions of that continuous variable. Within each bin, we calculated the proportion of model runs that met our minimum criteria for cows, crops, and woodlands for all 60 model years (were ‘sustainable’) and displayed this proportion as a line in our graphs. To examine how individual management interactions impacted sustainability and potentially interacted with rainfall scenarios, we divided a single rainfall scenario model result dataset into the different levels of each management intervention (e.g. stone walls or no stone walls) and binned them as before, creating two separate lines for the two different options for that management intervention within that rainfall scenario. Note that this averages over the other management interventions for that rainfall scenario; we do not examine interactions between all management interventions but only between the categorical ones and continuous ones (e.g. stone walls yes/no and proportion crops). In the figure captions, we report information on the number of model simulations that fall into these divisions of management options, rainfall scenarios, and bins of continuous variables.

With so many variables, it was impractical to test and interpret interactions of arbitrary order, so the graphical analysis helped to look for some of the potential interactions. The other disadvantage of the statistical model is that we have assumed a functional form: not only is that method incapable of demonstrating interactions unless they are explicitly included, but it also restricts the way in which we detect relationships between variables in general. We therefore use both methods (statistical and graphical) to evaluate model results, where possible cross-checking for consistency.

## Results

Below we review the results of validating the model against independent community-based data, the sensitivity analysis indicating whether underlying parameters had comparable effects to the management interventions and rainfall scenarios, and the impact of spatial configurations, management interventions, and rainfall scenarios on model sustainability.

### Validation of model

**Fig 4:**
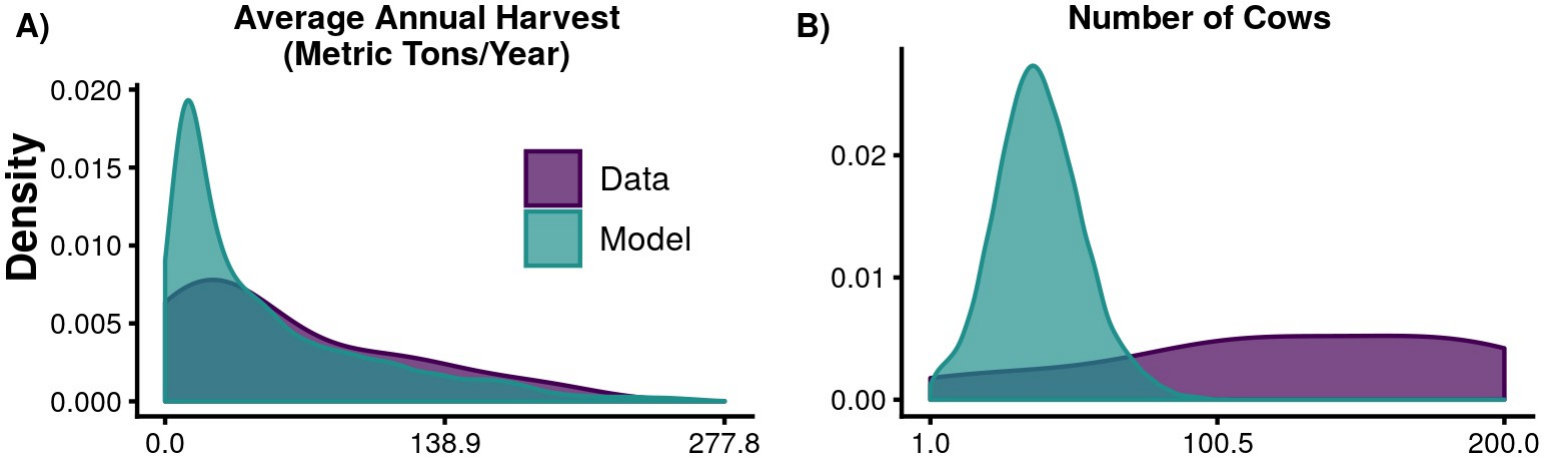
Comparison of field data and model outputs. Field-based data are shown in dark purple and model outputs are shown in light teal. A) Yearly harvest in metric tons (10% difference in the means, 9% difference in the standard deviations) and B) Number of cows (68% difference in the means, 76% difference in the standard deviations).

Comparing our ABM’s outputs for the parameters which reflect actual conditions over the last 60 years, we find that harvest values match somewhat well between field data and model outputs (10% difference in the means, 9% difference in the standard deviations), while cow numbers did not match very well (68% difference in the means, 76% difference in the standard deviations), though they do fall within the range of the field data (Fig 4). This comparison is based on the 316 of our 499,200 runs which used the ‘historical’ rainfall scenario, met the criteria of finishing a run (lasting all 60 model years), and had management parameters matching the actual historical management practices.

### Sensitivity analysis

**Fig 5:**
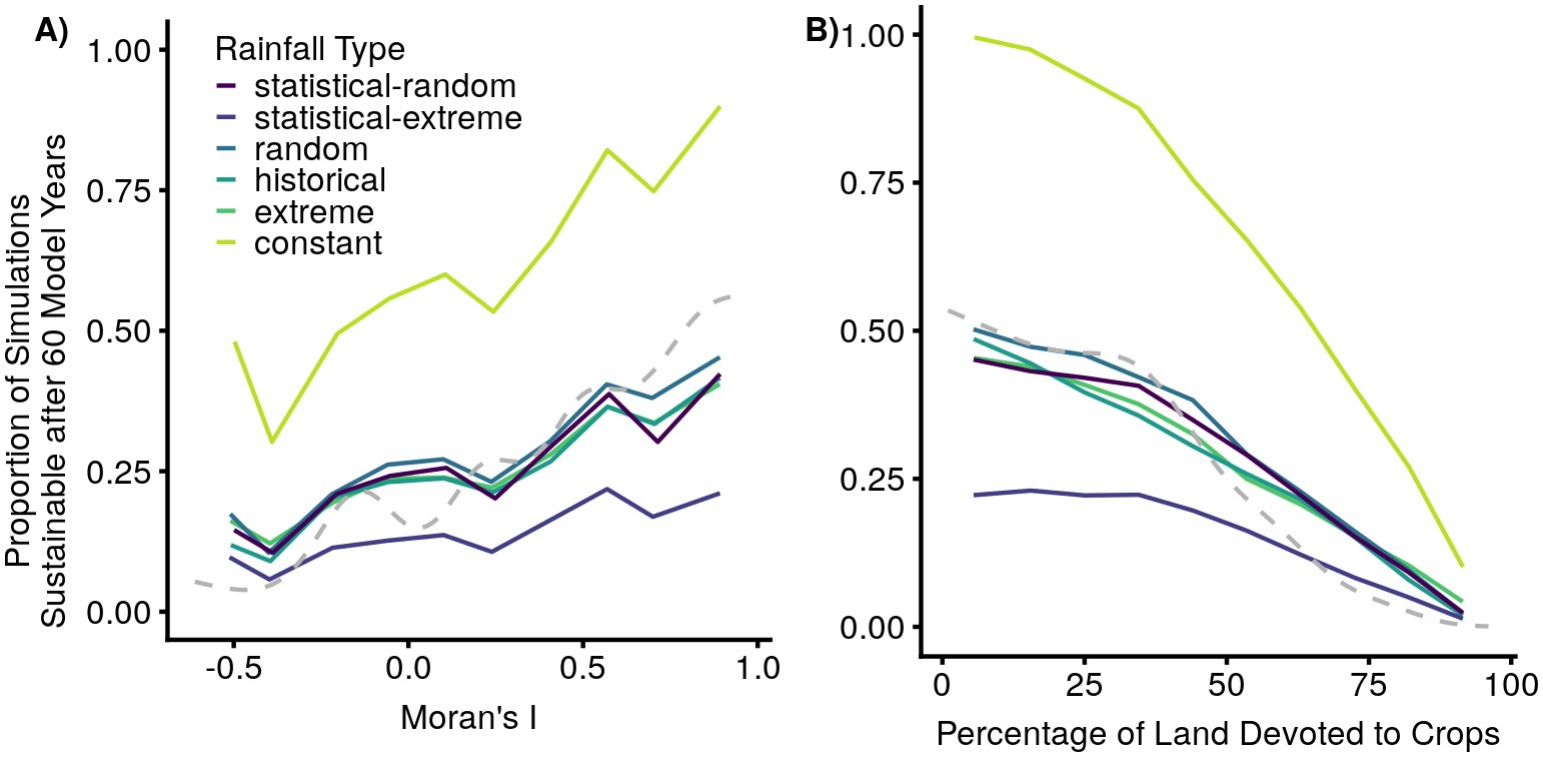
Model sustainability for different rainfall scenarios. Proportions of model runs that successfully met cow, crop, and woodland minima for all 60 model years (‘sustainable’), as influenced by A) spatial autocorrelation (Moran’s I, where negative numbers indicate anti-correlation similar to a chess-board, 0 indicates no correlation, and positive numbers indicate correlation) and B) proportion of land area dedicated to agriculture. The sustainability of the six different rainfall scenarios are shown in thick solid lines, calculated by binning the Moran’s I values or crop proportions (each divided into 10 bins, minimum N in each bin ranging from 520 to 8998 across different rainfall scenarios) and calculating the proportion of model runs that were sustainable, effectively averaging over all other variables. GAM smooth functions from the sensitivity analysis are shown as dashed gray lines.

The results of the GAM show that the rainfall scenario has the largest magnitude effect on the sustainability of a model run, and this variable was statistically significant (p<0.001). The categorical management interventions were close behind in magnitude (with p-values all <0.001). Of the underlying variables, only woodland growth rate had a magnitude similar to the management interventions (p<0.001). The other 18 underlying variables had smaller-magnitude effects and half of these were not statistically significant. Table 1 summarizes some sample variables from the GAM results, and S3 Appendix lists all parameter estimates, degrees of freedom, test statistics, and p-values. The overall model intercept was -1.67, which translates to successfully meeting minimum criteria for all 60 years 15.8% of the time (p<0.01). This represents the overall probability of a model lasting all 60 years, for a ‘base’ case: constant-rainfall scenario, no management interventions, 48% land use dedicated to crops, a Moran’s I of 0.28, and the values of all the underlying parameters as listed in S1 Appendix. We also show the GAM smooth functions for proportion crops and Moran’s I in Fig 5 for comparison to the graphical analysis.

**Table 1:** Sensitivity analysis results. Parameter estimates from the Generalized Additive Models testing the sensitivity of model outcomes to changes in various parameters. Each estimate reflects the change from a base case (model intercept) when A) comparing rainfall scenarios to the constant rainfall scenario, B) adding only one management intervention, or C) increasing one of the scaled, centered underlying variables by one unit from its mean value.

Independent Variable	Untransformed Parameter Estimate	Transformed Probability of Running all 60 Years	p-value∣
Base case (Model Intercept)†	-1.67	15.8%	< 0.001
**A) Climate Scenario Variables**‡			
“Random” Resampled Rainfall	-4.65	0.18%	< 0.001§
“Statistical-Extreme” Rainfall	-6.53	0.03%	< 0.001§
**B) Management Intervention Variables**			
Store Grain for 3 years	4.49	94.4%	< 0.001
Transport (subsidize) 70% of cows	2.11	60.8%	< 0.001§
Transport (subsidize) all cows	1.86	54.7%	< 0.001§
Move cows once a day	1.13	36.7%	< 0.001
Feed (subsidize) 70% of cows	1.12	36.5%	< 0.001§
Enhance growth of 10% of forest	0.888	31.3%	< 0.001
Feed (subsidize) all cows	0.872	31.0%	< 0.001§
Enhance growth of 10% of crops	-0.109	14.4%	< 0.001
Build stone walls	-0.248	12.8%	< 0.001
Smooth function of proportion crops	N/A	0.08–53.4%∣	< 0.001
Smooth function of Moran’s I	N/A	3.88–56.6%∣	< 0.001
**C) Underlying Model Parameters**¶			
Woodland growth rate (*g*_*wood*_)	0.147	17.8%	< 0.001
Catabolism efficiency(*η*_*c*_)	0.051	16.5%	< 0.001
Energy density of woodland browse (*η*_*browse*_)	0.045	16.4%	< 0.001
Chance cows don’t breed (*P*_*nr*_)	0.029	16.2%	< 0.001
Energy density of cow tissue (*η*_*cow*_)	0.015	16.0%	0.004
Total Mudhomori crop perimeter	-0.011	15.7%	0.032
Crop growth rate	-0.011	15.7%	0.031
Maximum cow mass (*m*_*max*_)	-0.017	15.6%	0.001
Minimum cow mass (*m*_*min*_)	-0.023	15.5%	< 0.001
Cow maintenance energy rate (*e*_*maint*_)	-0.061	15.0%	< 0.001

^∣^p-values adjusted for multiple comparisons using the “false discovery rate” method of [33]

^†^Base case for sustainability model: constant-rainfall scenario, no management interventions, 48% land use dedicated to crops, a Moran’s I of 0.28, and average values of all the underlying parameters. Similar for the annual harvest model.

^‡^Only largest and smallest rainfall parameters are shown.

^§^p-value for rainfall parameters is for all rainfall levels collectively, rather than individual levels/contrasts; the same is true for cow subsidy.

^∣^Ranges of probabilities of sustainable runs are shown for the smooth functions of Moran’s I and proportion crops.

^¶^Underlying parameters are only shown when their untransformed magnitude for the sustainability model is equal to or greater than those of the management parameters or if the effect was statistically significant at the p<0.05 level.

To give a better sense of the variables’ impact on whether a model run was likely to be sustainable, we transformed the parameter estimates to the probability scale; for example, storing grain for three years improves model sustainability from 15.8% in the base case to 94.4%, while resampling rainfall randomly from the historical time-series decreases model sustainability from 15.8% to 0.18%, compared to the base case of constant rainfall. By contrast, increasing (centered, scaled) woodland growth rate by one unit (corresponding to increasing woodland growth by approximately 6%) only increases model sustainability from 15.8% to 17.8%, and all other underlying parameters have effects smaller than that. Smooth functions of proportion crops and Moran’s I were significant (p<0.001). See S3 Appendix for parameter estimates, test statistics, degrees of freedom, and p-values for all variables tested.

### Rainfall scenarios

Constant rainfall models had substantially higher proportions of model runs that lasted all 60 years than any of the variable rainfall scenarios, including the actual historical time-series, while the ‘statistical-extreme’ models had the lowest proportion. This is true both in the statistical analysis (Table 1) and in the graphical analysis (Fig 5). There were slight differences between the other rainfall scenarios, but they were much more similar to each other than to the constant or statistical-extreme scenarios.

### Spatial configurations

**Fig 6:**
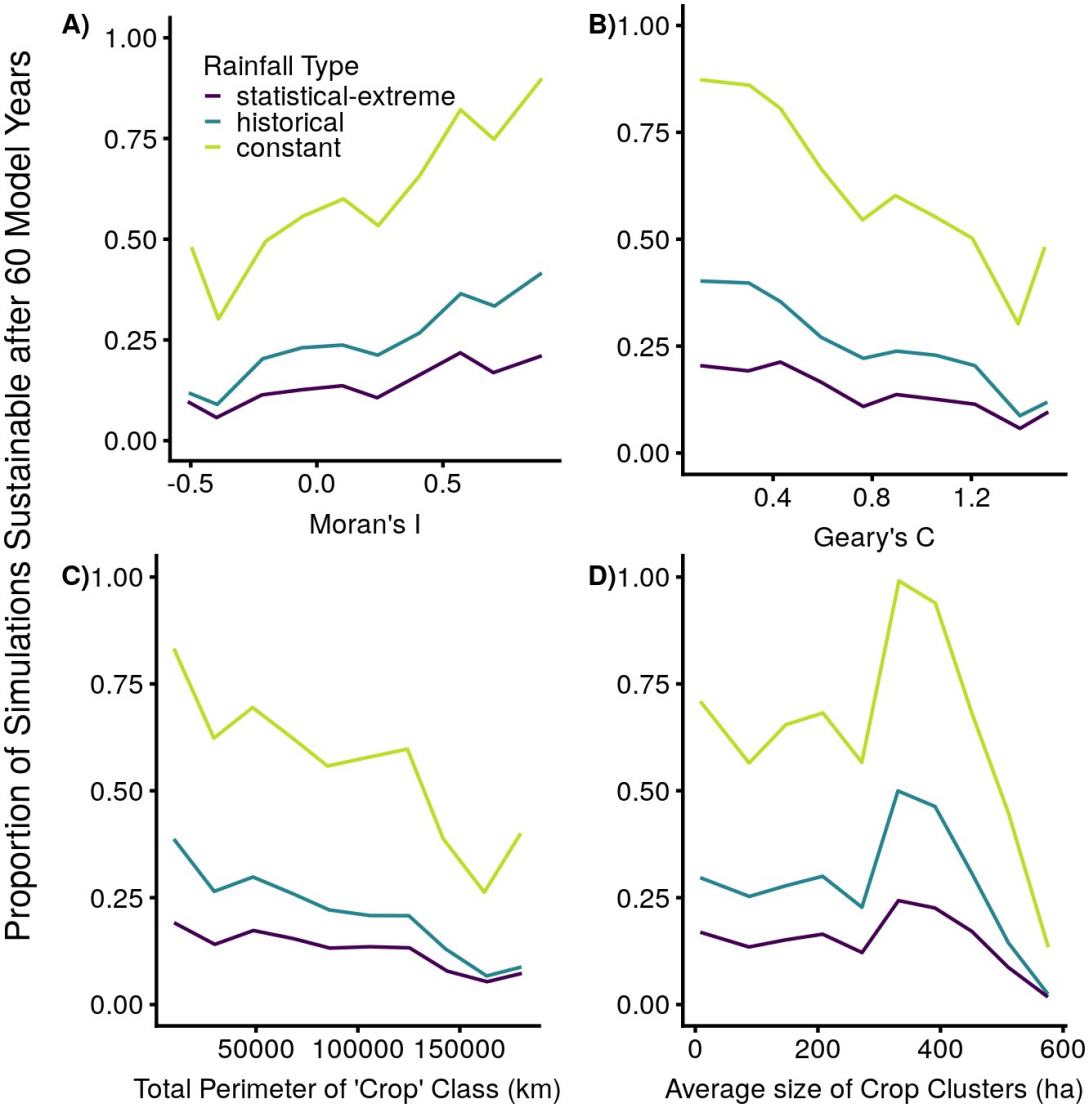
Model sustainability summarized by different spatial configuration variables. Proportions of model runs that successfully met cow, crop, and woodland minima for all 60 years (‘sustainable’), as influenced by A) Moran’s I (negative numbers indicate anti-correlation similar to a chess-board, 0 indicates no correlation, and positive numbers indicate correlation), B) Geary’s C (numbers below 1 indicate correlation and numbers larger than 1 indicate anticorrelation), C) the total perimeter of the ‘crop’ class in kilometers, and D) the average size of crop clusters in hectares. The sustainability is calculated by binning the spatial variables (each divided into 10 bins, minimum N in each bin ranging from 281 to 2901 across the three rainfall scenarios) and calculating the proportion of model runs that were sustainable, effectively averaging over all other variables. For clarity, only three of the rainfall scenarios are shown: constant, historical, and statistical-extreme.

Model run sustainability was approximately increasing with increasing spatial autocorrelation (larger clumps of crops/higher Moran’s I). Geary’s C [34] shows similar results, with large numbers (reflecting a more chess-board-like configuration) showing the lowest sustainability, and smaller numbers (reflecting a more clumped or homogenous configuration) showing higher sustainability. Model sustainability also generally decreases with increasing crop perimeter, while it has a more complex relationship with the average crop cluster size (Fig 6). Because these analyses aggregate over the range of proportion-crops, in S3 Appendix. we also show plots for three cases: 0-20% proportion-crops, 35-55%, and 80-100%. The pattern observed in Fig 6 is similar to the pattern in the low and intermediate case (0-20% and 35-55% crops), while there is little effect of spatial configuration for high levels of proportion-crops (80-100%).

### Management interventions

**Fig 7:**
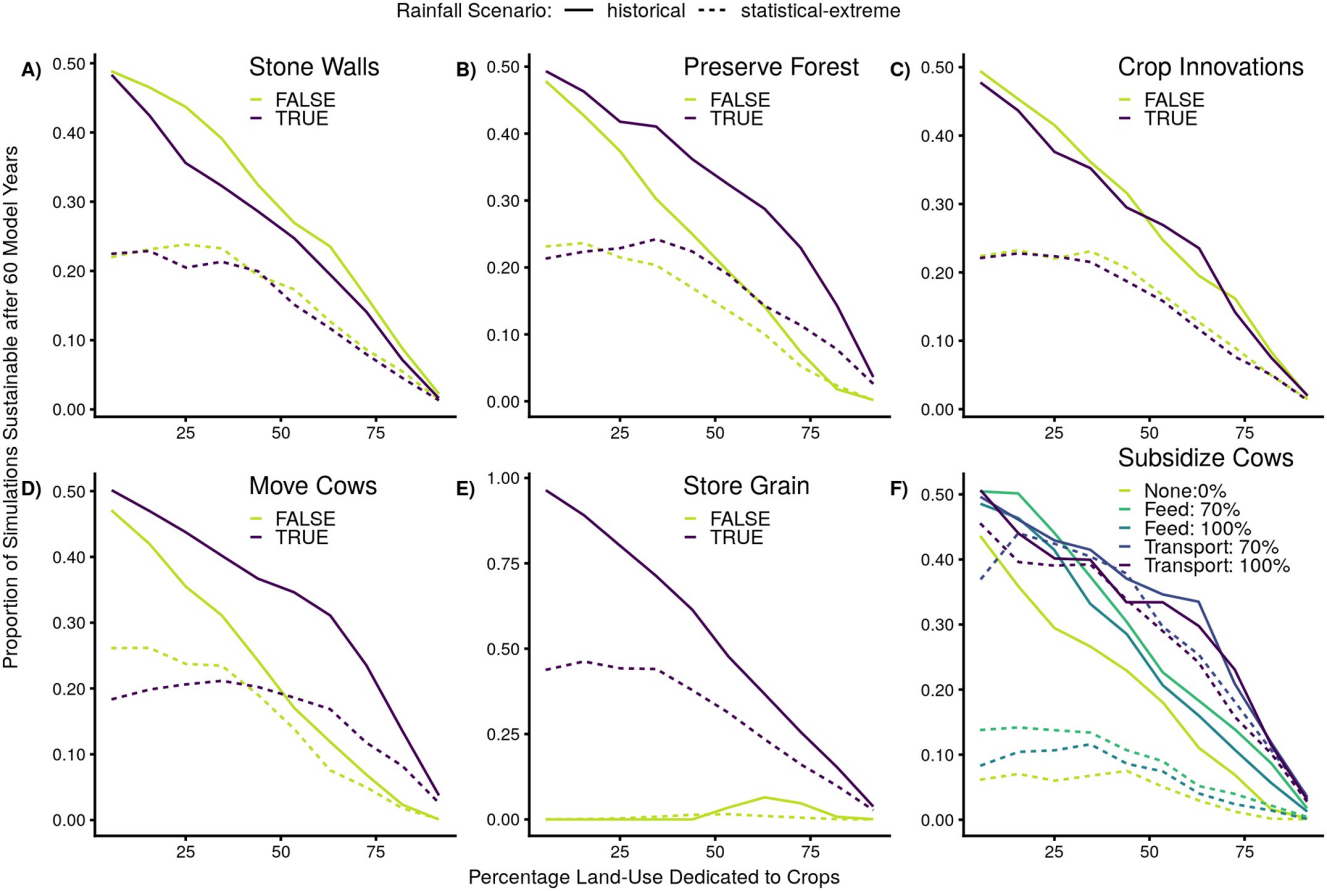
Model sustainability for different management interventions. Proportions of model runs that successfully met cow, crop, and woodland minima for all 60 years (‘sustainable’), as influenced by proportion of land dedicated to crops, rainfall scenarios, and management interventions. Models using the historical rainfall time-series are shown in solid lines, and ‘statistical-random’ rainfall scenario models are shown in short-dashed lines. Management interventions are: A) building stone walls, B) preserving forest, C) introducing crop innovations, D) moving cows to better grazing, E) storing grain for three years, and F) subsidizing cows by feeding them or transporting them completely outside the village. Lines are generated by binning the proportion of crops (10 bins, minimum N per bin ranging from 1676 to 4542 across the combinations of rainfall scenarios and management options) and calculating the proportion of sustainable model runs in that bin for that combination of rainfall scenario and managment intervention (averaging over other management interventions). See S3 Appendix for similar figures representing the effects of rainfall scenario and management intervention for binned values of Moran’s I and other spatial configuration variables, as well as for other rainfall scenarios.

Management interventions as implemented in the model varied in their ability to affect sustainability (Fig 7). Model run sustainability was approximately decreasing with increasing proportions of land area devoted to crops, given the biologically minimal thresholds we used (Fig 5). Storing grain greatly improves the sustainability of model runs, and to a lesser extent, transporting cows out of the system, preserving forest, and moving cows to better grazing do so as well, while stone walls and crop innovations have less of an impact. These graphical results match the sensitivity testing (statistical) results, and are similar for different rainfall scenarios and spatial configuration variables (See S3 Appendix).

There are several potential interactions between our variables. Subsidizing the cows by transporting them (Fig 7F) gives a much bigger increase in sustainability for the statistically extreme rainfall scenario: sustainability is very low in that scenario when cows are not transported (light green dashed lines), but when they are transported (blue and purple dashed lines), the sustainability is similar to the other rainfall scenarios. In addition, there appears to be potential for an interaction between the proportion of land dedicated to crops and moving cows, and between proportion crops and preserving forest: these management interventions are more effective at intermediate proportions of crops.

## Discussion

Our model of the complex agro-pastoral system in Mazvihwa captures the basic dynamics between livestock, woodland grazing land, and arable production. Below, we discuss what validation was possible for this model; the issues of model complexity, realism, and fidelity; and our use of sensitivity analysis in participatory modeling. We also discuss some advantages and disadvantages of our statistical and graphical analysis as well as our ways of representing spatial configurations and rainfall variation. Finally, we discuss a number of complex-system behaviors demonstrated by our model, including the importance of feedbacks, the effects of exacerbating versus mitigating variation, and the tradeoffs in emphasizing one system component over another.

### Validating a model based on heterogeneous community data

The validation of complex simulations (whether community-based or not) is an essential aspect of their use in understanding the systems of interest. [35] In the context of our participatory modeling project, there are three different ways we are discussing model validation. First, the sense of structural or ontological validation: that enough of the right elements are present in the model with the correct mechanisms driving them. [36] Second, the evaluation of the model as useful and credible to the user group. [10] Both of these are typical of participatory modeling, but in our case, because the community had more quantitative data, we explored a third type of validation: behavioral validation, comparing the model’s quantitative output with the community’s field data. [17]

Our structural and practical validation were quite successful; however, our behavioral validation results are mixed. The agreement between the simulated and actual harvest numbers is reasonable, but the agreement between the simulated and actual cow numbers is not. Our coarse calibration step encouraged the simulated cow numbers to be within the range of the data, but the shape of the distributions is quite different. This difference may be attributable to the fact that we only modeled the natural dynamics of cows, while in reality cows are seen as a sign of wealth so farmers use many more strategies for increasing their numbers than we were able to feasibly include in our model. The order-of-magnitude agreement of our other three variables (minimum woodland, subsidy spent, and crop eaten) is encouraging because those numbers were not used in any way in the calibration process. Though our cow numbers are not well-validated, we continued with our sensitivity analysis because we felt it was important to evaluate whether underlying parameters had impacts as large as our management interventions and rainfall scenarios (which were the processes of interest to the community).

This multiple use of validation is not unusual for many modeling disciplines: Systems dynamics modelers, for example, have long engaged in similar processes of integrated quantitative and qualitative validation of simulation models. [37] Participatory modeling projects already often use quantitative and qualitative data, [8, 38] but will also be in a position to use quantitative and qualitative validation processes more and more, as citizen science projects yield larger community-held datasets. Communities may begin to consider classic techniques such as dividing datasets into ‘training’ datasets for calibration and ‘test’ datasets for behavioral validation. Where model and actual time-series can be directly compared, a wide variety of techniques can be applied, including cross-correlation. [39] Calibration of parameter values could be determined using algorithmic optimization. [16] These practices still only work for model behaviors within the range of historical data, of course, and the validity of underlying processes (structural validation) will still be the best test of whether the model’s predictions into novel situations are believable.

### Model complexity, realism, and fidelity

We raise the question of simplicity versus complexity because there are challenges associated with analyzing complex models, and because ecological and statistical modelers have debated the relative merits of different strategies regarding model complexity for decades. Simpler models may be used to generalize and inspire questions for further investigation, while more complex models may be used to represent a particular system and help make management decisions for it [40]. Some generalized, mechanistic models might be criticized as oversimplications, but making mechanistic models more representative of the world tends to make them more complex (e.g. Global Circulation Models for studying climate; [41]), and there is a tradeoff between the complexity of the model and its interpretability or practical use. Some authors suggest that there is an “optimal zone of model complexity” [27] for a given task. Our model certainly uses many simplifications of the system, but is still complex enough that interpreting its results can be difficult. For example, interpreting the statistical results of a GAM with so many variables is challenging even though it only includes main effects and no interactions; and graphically evaluating results for different management interventions while stratifying by different rainfall scenarios is equally difficult: even with almost 500,000 simulations, it is still possible with a narrow enough selection of input parameters to have an N small enough that a proportion calculation is biased by the small sample size.

Our model complexity (i.e. what details to represent and what to simplify) was guided by the community-based structural validation. In particular, once the model was realistic enough to be used effectively as a discussion tool with other community members and local leaders, we did not attempt additional model behavior complexity. Rather, at that point, we focused on biomass accounting and refining specific parameter values via literature review, in order to attempt quantitative behavioral validation of model outputs against field data. This resulted in a variety of simplifications. Crop biomass, for example, was treated in an undifferentiated fashion, despite the differences in drought tolerance between maize and native varieties of small grains (sorghum and millet). We do reference this difference obliquely, in that the ‘crop innovations’ management intervention allows crops to grow faster; we intended this to refer to a variety of Muonde Trust agricultural innovations including encouraging farmers to grow drought-tolerant native varieties of crops, though this is grouped with other Muonde innovations like water harvesting techniques that improve infiltration and reduce runoff. The simplification of increased variation due to climate change into a year-to-year change in rainfall was another choice which was made in trading off between model development time and sufficient realism for the model to be a useful discussion tool; though floods and droughts and other within-year rainfall variation can be more damaging, the community was able to discuss these issues inspired by the increased year-to-year variation that was included in the model. We also considered doing another round of modeling following community workshops in which we could incorporate their choices during the computer-mediated role-play into a model with explicit farmer agents, but this was also beyond our scope. Not all of our simplification choices were logistically-based, however. Though our model is spatially explicit, it is not spatially realistic. [20] We chose not to represent the actual crop and woodland configurations in Mudhomori because too much spatial realism can hamper the use of an ABM as a discussion tool for generating “innovative and integrative solutions.” [42] Finally, we note that we have made the model specific to cows, when we could have modeled abstract livestock. In this case, cows were chosen due to their cultural importance, so this is an example of making the model more legible to the community by being more specific and making the model less generalizable for outside audiences.

At a higher conceptual level, we can also break down how faithful our model is to various components of the agro-pastoral system. Models of complex social-ecological systems can be evaluated by the level of fidelity of each of their components, for example economic, ecological, policy, and human behavior aspects. [43] In our model, we have moderate ecological fidelity: specifically, we carefully account for ecosystem-level energy and biomass flows, while having low species-level fidelity (woodland biomass is undifferentiated, as is crop biomass), and while we do have a two-stage population model for cows, it is not sex-differentiated. We do include the price of feed used to supplement livestock diets, but this price does not respond to demand or supply, so the model has low economic fidelity. Human behavior is moderately faithful to how humans behave in the real system: farmers move cows from day to day, subsidize in some years and not others—however, they do not adapt these strategies over time as real farmers do. Also, because we have only represented a fraction of the real farmers’ management strategies and have not differentiated between the roles different humans play in each of these decisions and management actions (instead just calling them all ‘farmers’), this points towards low-to-moderate fidelity regarding human behavior. Though no one component has particularly high fidelity, we have represented a wide range of key aspects of the system somewhat faithfully, and in particular, faithfully enough for the community to find the model recognizable and therefore useful. Our additional quantitative calibration and behavioral validation sets our model apart from other Zimbabwean agro-pastoral ABMs: there are models which differentiate human actors into herders, farmers, and so on but do not use realistic parameters [19] or model economics more carefully and allow land use to change but do not model the interactions of livestock with landscape management. [18] Of course, each modeling team has their own priorities regarding how to construct and validate their representation of the system.

### Sensitivity analysis for a complex community-based model

Sensitivity analysis can be used to determine what elements of a model can be removed. Our structural validation process gave rise to a model in which community members had determined that entities, behaviors, and parameters needed to be included. Therefore, we employed sensitivity analysis only to determine which parameters have the most impact on the model outputs, as opposed to being used to determine if a parameter or other element can be “fixed” (set constant) [44] or dropped altogether. [16] In this case, none of the parameters could be ‘dropped’ if the sensitivity analysis indicated that they were not important: because the model was built up from simple to complex, parameters and behaviors were only added when deemed necessary. We do acknowledge that this locks in “path dependence” in terms of the order in which we added model elements; if we had added them in a different order, different components might have been deemed necessary in order to structurally validate the model. [45]

There was also a practical consideration in our choice to only do a local sensitivity analysis: our model construction and calibration methods resulted in a model with a high-dimensional parameter space. Global sensitivity analysis is sometimes recommended for ABMs, [13] but our model’s complexity meant that exploring the entire space (range of all possible values for all parameters) was infeasible even with high-performance parallel computing resources. In addition, doing a global sensitivity analysis on all numerical parameters would likely reveal many local minima where the analysis would give totally different results for some parameters. Therefore we chose to calibrate the model’s parameters to data first, and then test the local sensitivity of the parameters once the variables were in the neighborhood of realistic values. Even with only local sensitivity tests, the number of parameter combinations we wished to test made high-performance computing a necessary part of our process. Due to this already-large number of model runs required, we made some additional logistical choices not to test the sensitivity of two model important model aspects: initial conditions and stopping criteria. With respect to initialization, we made specific choices in our initialization strategy to get the modeled system as close to equilibrium as possible before beginning rainfall variation simulation (see S1 Appendix). And though we know they are extremely important in determining model results, the sensitivity test to stopping conditions (sustainability thresholds) was simply beyond the scope of this study and was left for future analysis.

Sensitivity analysis is also often used to highlight parameters that should be better known and to provide caution where they are not. Our analysis showed that rainfall scenarios had the biggest impact on model sustainability, followed by management parameters, and then by underlying parameters. This suggests that despite some underlying parameters being less certain or coming from literature not based on the study system, the model does what it was intended to do: explore the relative impacts of management interventions and rainfall scenarios. The model results’ sensitivity to rainfall variation implies that this is indeed a key aspect of the system to model more realistically in future work. The only important underlying parameter was the woodland growth rate, which did have a magnitude similar to a few of the management interventions (enhancing crop growth and building stone walls). This implies two important points: first, the results for those management interventions should be understood with the caveat that they are conditional on a faster or slower growing woodland biomass, because their magnitudes are similar, and second, if the community wishes to focus on answering their questions more precisely using the model, measuring woodland growth more thoroughly could be an important goal. Alternatively, a later iteration of the model might make the woodland ecology more sophisticated in order to understand what aspects of the ecology are as important as some of the management interventions. That said, most of the management interventions had a much larger impact so we feel comfortable discussing them with respect to the management of complex systems.

### Technical comments on analysis methods, rainfall scenarios, and spatial configuration variables

There were advantages and disavantages to the statistical and graphical analysis methods, but we ultimately found them to be complementary in assessing our model’s results. The GAM smooth functions control for the other variables (conditioning on specific values), while the proportions displayed in the graphical analysis average over all the other variables and only stratify by rainfall type (marginalizing over the other variables). The GAM smooth functions sit well below the constant rainfall solid line (Fig 5) because the solid line includes the effects of some management interventions which the smooth function does not (most notably, half of the simulations use the grain storage intervention, which has a strong positive effect, and the GAM base case does not include this). The shapes are somewhat similar, however, with the sustainability declining as proportion crops rises, and roughly increasing as spatial autocorrelation increases. The statistical analysis is able to control for proportion crops when it estimates the effects of spatial autocorrelation, and vice versa, without triggering the issues of small sample size in a given bin (see S3 Appendix). The graphical analysis, however, allows us to see the potential for interactions between rainfall scenarios and management interventions (keeping in mind that they still average over the other management interventions), which the GAM does not include. Using both methods together helped us to understand the behavior of our model.

We found representing the spatial configuration of the system with a single variable to be challenging. Moran’s I and Geary’s C, while classic measures of spatial autocorrelation, both have a theoretical problem in our case: because we have only two classes, these variables group together configurations with either a large quantity of crops or a large quantity of woodlands. This may explain some of the oscillation in the smooth function of Moran’s I produced by the GAM. The other two landscape ecology variables are defined based on the ‘crop’ cover class, so they are unique across all proportions of crops rather than combining results from high proportions and low proportions. Total crop perimeter has the simplest relationship: model sustainability generally decreased as the crop perimeter increased, likely due to increasing need for fencing material (and greater cost to woodland biomass) and a longer border affording easier access for cows to break into crop fields and reduce harvest. On the other hand, the average crop cluster size had a more complex relationship with sustainability. One reason for this is the confounding of these spatial variables with the proportion of land dedicated to crops: the patterns of model sustainability versus crop cluster size were particularly different for different proportions dedicated to crops (S3 Appendix).

Lastly, we discovered that there was little difference between parametric and nonparametric bootstrapping of rainfall time-series. The modeled system’s sustainability mainly responded to the amplitude in the rainfall variation rather than how the amplitude of the variation was created. This observation largely held for all continuous variables and management interventions and was true in both the graphical analysis and the statistical analysis. This behavior is consistent with the idea that larger oscillation in the system’s driver may lead to larger oscillations in the system’s behavior (in this case resulting in cow population explosions or crashes). Of course, a more detailed within-year model for increasing rainfall variability could reveal different results; however we feel it is useful to make the note that, at least for our model, it was the range in the variation rather than the method of generating the variation that mattered most.

### Behaviors of a complex agro-pastoral system

Due to latencies and broken feedbacks, managing for one component of the system may come at the expense of the other components. Some combinations of interventions actually worsen our model’s ability to persist all 60 years, while at the same time increasing either harvest or livestock numbers. For example, crop innovations and stone walls both increase average annual harvest at the expense of the sustainability of the model system. The stone walls, while protecting harvest from cow consumption, may be so effective that cow populations crash in low-rainfall years because they lack an additional food source. At a larger scale, state interventions into social-ecological systems can decouple the local people from their ecosystems, with detrimental effects for the system’s resilience, [46] so at the scale of Mazvihwa’s system, decoupling system components could also decrease the system’s resilience. Tight feedbacks keep managers accountable for the impacts of their management choices. [1, 47]

At the same time, interventions like feed subsidy that help cow populations survive in bad years can be problematic as well, especially when rainfall is more variable, because breaking the shorter term feedback loops between forage and cow populations means their populations can grow so large that longer term feedback loops cause population crashes; this behavior is seen in other models of complex systems where the introduction of outside subsidies into the system can be destabilizing. [48, 49] This behavior is not as problematic when subsidizing by taking the cows out of the system (‘transport’ subsidy), likely because the cows that are transported are not also consuming woodland resources at the same time as their population is being increased by subsidy, and our implementation reduces the reproductive capacity of transported cows relative to those still in the system. Of course, in the real system the transported cows are consuming some other ecosystem’s resources; however, this resembles grazing rotations which can decrease pressure on any one part of an area and increase beneficial ecosystem heterogeneity. [50] Even without subsidy, because there is a latency in cow population growth relative to the rainfall and woodland growth (due to the maturation time of calves), these two components can become decoupled and allow cow populations to become too large.

On the other hand, interventions which smooth over year-to-year variation, especially those that act on outputs of the system rather than feedbacks between system components, are extremely helpful in ensuring system sustainability. The effect size of the storing grain intervention has a similar value to the difference between constant rainfall and randomized rainfall. Storing grain has the effect of smoothing over variability in rainfall in both the model and the real system, allowing bumper crops from a good year to carry the system through a series of bad years, and this has been an extremely effective strategy for the farmers in the real system. In reality, the crop innovations of the Muonde Trust include water harvesting practices that can smooth over rainfall variability by increasing groundwater infiltration; however we did not model this subtlety.

## Conclusions

ABMs have been challenged to “demonstrate that they can solve problems in the real world better than traditional modelling approaches.” [51] In participatory contexts, with appropriate structural validation, simulation models can be strongly grounded in “what is” and therefore more powerful to “explore pressing, but otherwise unapproachable questions of ‘what if?’” [52] Through our modeling process, we were able to create a tool which helped the Muonde research team to discuss the consequences of current land-use policies. Since the model’s creation, Muonde has been able to use it to convene local leaders around the issue of how much land is used for arable production. New policies developed by Muonde and these leaders allow fallow fields to be re-cultivated rather than cutting down woodland grazing area, and Muonde is currently piloting these new strategies in Mudhomori and nearby villages. If the most important criterion for community-based model validation is its use to users, [10] then our model was successfully validated in that sense.

Future work with this model in its current form could examine more closely how the definitions of sustainability change the results: as an example, the impact of proportion-crops would be quite different if a higher harvest threshold were required. In that case, low proportions are not sustainable (too little harvest), and high proportions are not sustainable (not enough cows or woodland), and the sustainability curve would become bell-shaped rather than monotonically decreasing as it did in our analysis here. In addition, investigating the response of the modeled system to a wider range of rainfall variation scenarios might reveal how the complex system responds to the underlying drivers of change. And of course all kinds of additional sophistications could be introduced into the model’s structure, including variation or adaptation of strategies over the 60 years, differentiating types of crops or woodland, or additional detail on the economic aspects of the system. Finally, future work could examine how the participatory modeling process supported the local-level policy change in Mazvihwa. We believe our model shows the potential promise of multiple forms of validation for participatory modeling as communities become better endowed with the data necessary for these methods.

## Supporting information

S1 Appendix Data and model details. Contains a detailed description of all data sources and calculations underlying model parameters, including tables of numerical data used to estimate parameters and for validation calculations.
(PDF)

S2 Appendix Details of technical implementation. Different model modes, details of software unit tests, profiling, and behavior tests, how we perturbed parameters for sensitivity analysis, how we tracked NetLogo patches and agents for better model updating efficiency, spatial and temporal scale restrictions of the model, details of creating crop spatial configurations and other initialization, notes about some of the updating procedures, and an outline of the order in which model functions (NetLogo procedures) are called.
(PDF)

S3 Appendix Full tables of sensitivity analysis and additional results figures. Parameter values, degrees of freedom, test statistics, and p-values from generalized additive statistical model for sensitivity testing of agent-based model results; and additional results figures.
(PDF)

S1 File R scripts to create publication figures and run statistical analysis of simulation outputs. (R)

S2 Dataset The model sensitivity test parameter sweep results—validation. Yearly values for model runs matching historical management and rainfall parameters (316 runs), used for validation comparison figure.
(CSV)
