# Supplementary material for: Using mixed methods to construct and analyze a participatory agent-based model of a complex Zimbabwean agro-pastoral system
Source: PLoS One. 2020 Aug 21;15(8):e0237638. doi: 10.1371/journal.pone.0237638 (PMC7442250; doi:10.1371/journal.pone.0237638)
Supplement: S1 Appendix — Contains a detailed description of all data sources and calculations underlying model parameters, including tables of numerical data used to estimate parameters and for validation calculations. (PDF) [file pone.0237638.s001.pdf]

S1 Appendix for: Using mixed methods to construct and analyze a participatory agent-based model of a complex Zimbabwean agro-pastoral system. [Data and model details.]

M.V. Eitzel[1*], Jon Solera[2], K.B. Wilson[3], Kleber Neves[4], Aaron C. Fisher[5], André Veski[6], Oluwasola E. Omoju[7], Abraham Mawere Ndlovu[3], Emmanuel Mhike Hove[3]

1 Science and Justice Research Center, University of California, Santa Cruz, Santa Cruz, CA, US
2 Seven Points Consulting, Lafayette, CA, US
3 The Muonde Trust, Mazvihwa Communal Area, Midlands Province, Zimbabwe
4 Universidade Federal do Rio de Janeiro, Rio de Janeiro, Brazil
5 Lawrence Livermore National Laboratory, Livermore, CA, US
6 Tallinn University of Technology, Tallinn, Estonia
7 National Institute for Legislative and Democratic Studies (National Assembly), Maitama, Abuja, Nigeria

* mveitzel@ucsc.edu

Model data sources

This model's parameters and behaviors integrate a variety of data sources regarding the system of interest. These include community-sourced quantitative and qualitative data from the Muonde Trust research team, as well as rainfall data from the Zimbabwean government and parameters drawn from the literature. See Table 1 for the values of specific model parameters, and the next section on "Calculations and processes incorporated in NetLogo model" for additional calculations associated with these parameters.

Community-sourced data and research methodology

The Muonde team has used a variety of methods to study their agro-pastoral system during the 1980s through mid 2010s, including structured and unstructured interviews with farmers, field measurements, and estimates from aerial imagery. We outline the specifics below.

The team has been tracking a set of sample households throughout Mazvihwa since the 1980s, interviewing farmers and monitoring crop yields, livestock numbers, and a variety of other aspects of the agro-pastoral system. The household sampling is as follows.

In the initial 1980s Mazvihwa-wide sampling, one representative "focal" household in each village of Mhototi Ward was chosen with the guidance of the late Ward Councilor, Mr. Bwoni, and late local Headmaster Mr. C.G. Mukamuri. Individuals in the focal household were then asked what other households they worked intimately with, creating a larger cluster to avoid the problems associated with studying households in imagined isolation. These judgments of household-to-household interaction were also confirmed with other members of the community. Sharing of cattle was determined to be the most significant measure of interaction, so field observation of cattle sharing was used to confirm what respondents claimed about their connections with other households. This

Table 1. Model Variables.

NetLogo Variable Name (Symbol)	Units, Values, & Data Sources	Explanation
**Spatio-temporal (scaling) variables**		
world-size	50 (unitless) †	Linear dimension of the village in number of Netlogo patches.
ha-per-patch	0.24 ha/patch ‡§	Mudhomori is 600 ha in size: 600/ (world-size*world-size)
ticks-per-day (t_{day})	3 ticks/day †	Number of time steps per day. (1 tick = 8 hours)
ticks-per-year (t_{yr})	1095 ticks/year §	365 * ticks-per-day (must be integer)
calendar-year	1952-2011 ‡	The water-year: July 1st of that year through June 30th of the next year.
**Cow-related (agent) variables**		
Hours-to-plough-ha (h_{work})	8 cow-hours ‡	How many cow-hours to plough a hectare.
max-cow-mass (m_{max}), min-cow-mass (m_{min})	63-296 kg [1]	Range of values for cow mass.
calf-birth-mass (m_{min})	18 kg [2]	How big a calf is at birth.
cow-maintenance-energy-rate (e_{maint})	980 kcal/kg/day	How much energy a cow burns by metabolizing and moving.
cow-working-energy-per-hour (e_{work})	920 kcal/hr [3]	Energy required to plough a field.
production-efficiency (η_p)	0.49 (unitless) [4]	Efficiency of turning energy into more cow mass.
catabolism-efficiency (η_c)	0.9025 (unitless) [4]	Efficiency of turning cow mass back into energy.
kcal-per-kg-of-cow (e_{cow})	1360 kcal/kg [5]	Energy density of cow when metabolizing.
livestock-not-reproduction-rate (P_{nr})	0.75 (probability) ‡	Probability per year of a cow not reproducing.
kcal-per-kg-of-browse (e_{browse})	1931 kcal/kg [3]	Energy density of woodland plants.
kcal-per-kg-of-crop (e_{crop})	2325 kcal/kg [6, 7]	Energy density of crops.
cost-of-supplemental-feed (c_{crop})	14.50 USD / 50 kg ‡	How much supplemental feed costs.
**Crop- and woodland-related (patch) variables**		
zero-crop-growth-intercept	232 mm ‡	Minimum rainfall for crops to grow.
crop-growth-slope	0.8731 kg/ha/mm/yr ‡	Amount that the crops grow per mm of rainfall.
woodland-growth-slope (g_{wood})	6.26 kg/ha/mm/yr ‡, [8]	Growth function for woodland – how much does the woodland grow per mm of rainfall.
muonde-efficiency	3.0 (unitless) ‡	How much faster crops grow when Muonde projects have been adopted.
**Fencing-related variables**		
total-mud-crop-perimeter	50,000 m ‡	Total length of fence in Mudhomori.
wood-to-build-fence-per-meter	5.77 kg/m/year ‡	Amount of woodland biomass needed to build one meter of fence.
termite-activity	2/3 (per year) ‡	Proportion of the fence lost per year to termite consumption.

†Spatial and temporal resolution established so that cows move realistic linear distances and plough a realistic area in a single tick.
‡From community-based data collected by the Muonde research team.
§Calculated.

procedure defined a set of initial households for the survey. Households were added to the cluster when a current or former member of the cluster established a new rural household as household head in a new physical space, or a member of the patrilineage of the nodal household returned in the face of divorce, job loss or other hardship. Where one individual took over a household in the same physical space (e.g. a widow from her husband) we considered that to be the same household.

For this model, we have focused on one village, Mudhomori, and scaled up values for the sample households in that village up to the entire village. Sample households initially comprised 20% of Mudhomori's population in the 1980s, and this proportion was verified in the 2010s by counting households on Google Earth; the proportion had changed less than one percentage point so we continue to use the same 5x multiplier.

Mudhomori village was estimated to be 600 ha in size based on estimation from aerial imagery. Percentages of village area classified as woodland grazing area versus arable crop field area are given in Table 2.

Table 2. Area dedicated to arable crop production. Estimated from aerial imagery and via field visits and interviews with farmers.

Year	village woodland area (percent)	village field area (percent)
1986	58	42
1991	53	47
1992	53	47
1993	53	47
1999	55	45
2007	45	55
2009	41	59
2010	41	59
2012	37	63
2013	35	65

From structured interviews with Mudhomori farmers, the Muonde team recorded yearly yield data for cereals and pulses in kg/ha from 1984-1986, 1990-1992, 1994, 1997-1998, and 2007-2011 (Table 3). This was recorded during harvest when farmers help each other count 'bags' of known weight (in kg). For the purposes of validating our model, we combine the harvests of different kinds of crops into a single biomass. Historical (1980s) and recent (2010s) proportions of crop and woodland by land area were estimated from aerial imagery, with extrapolation in intervening years from measured field areas of sample households.

Also from structured interview data, the Muonde team recorded yearly livestock numbers (cattle, donkeys, goats and sheep) in 1986, 1991-1993, 1999, 2007-2010, and 2012-2013 for a subset of sample households and then extrapolated up to the whole village (Table 4). Farmers reported reproduction rates of cattle in good (high-rainfall) and bad (low-rainfall) years. Interviewees also quantified damage when livestock break through fences and consume crops (reporting the number of plants eaten by several different kinds of livestock). The team also obtained estimates of the number of hours it takes an animal to plow a hectare of cropland. Finally, farmers reported whether they used supplemental feed in low-rainfall years, and gave market rates in US Dollars per kilogram for the feed in 2015-2016. We use constant 2015/2016 dollars in both the model and the validation calculations.

The Muonde research team measured the length of all the fences in Mudhomori in 2013 by pacing after training against a meter ruler (i.e. measuring stride length). For a given length of fence of a type typically used in Mudhomori village, they weighed the

Table 3. Agricultural production data. Measured during harvests for sample households, usually in a collective and semi-ceremonious way as neighbors and relatives help each other and count quantities in "bags" and other units. The size of the bag unit has altered over time between 118kg, 90kg and 50kg. Field researchers evolved with local designation, clarifying with the farmers when necessary regarding the unit of measure. Numbers are averaged per household.

Year	Cereal Yield/Ha	Pulse Yield/Ha	Total Yield (Food Crops) in Kilograms/Ha
1984-85	767.27	16.42	783.69
1985-86	86.35	3.99	90.34
1986-87	72.74	14.08	86.82
1990-91	0.00	0.00	0.00
1991-92	0.00	0.00	0.00
1992-93	380.43	0.66	381.09
1994-95	41.52	9.44	50.96
1997-98	73.88	20.41	94.29
1998-99	369.81	90.22	460.02
2007-08	72.41	43.25	115.67
2008-09	247.31	71.22	318.53
2009-10	117.99	54.36	172.36
2010-11	124.88	29.71	154.59
2011-12	29.21	4.16	33.36

Table 4. Counts of cattle for sample households. these counts were multiplied by a factor of 5 to scale up to the whole village. This gives a range of 5-200 cattle, which we use to compare for validation of the model outputs.

Year	Cattle
1986	20
1991	20
1992	7
1993	1
1999	18
2007	40
2008	36
2009	35
2010	39
2012	26
2013	28

amount of brushwood (*matyatya*) and fence poles using a 25kg salter scale with 100g increments. In addition, the team recorded the rate of fence replacement, by noting when fences of known age needed to be rebuilt due to termite damage.

Woodland growth was measured for two dominant tree/shrub species: *Acacia tortilis* and *Colophospermum mopane*. Trees were cut, and then after a set time interval, they were cut again, and the new biomass air dried and weighed to determine accumulation over time (Table 5). Acacia data were collected by the Muonde research team in 2013, and data for mopane growth were collected by Philemon Ndumo and others now associated with Muonde Trust in 1988 under a project supported by ENDA-Zimbabwe with Ford Foundation funding using the same methodology of cutting and drying; data were presented to ENDA by I.C. Scoones and later re-analyzed by K.B. Wilson. The Muonde team estimated the proportion of the woodland that supports trees from aerial imagery, as well as the relative proportions of mopane and acacia (verified with

field-based visits and interviews with farmers).

Table 5. Woodland growth data.

Months Since Cutting	Wet Weight/Kg/m2	Dry Weight (Kg/m2)
Mopane:		
17	0.595	0.2975
28	0.785	0.3925
29	0.805	0.4025
41	1.2	0.6
53	1.125	0.5625
76	1.615	0.8075
Acacia:		
12	0.49	0.24
28	2.32	1.51
36	1.43	0.90

Qualitative data via open-ended interviews on topics of agro-pastoral production and stewardship yielded rich information on cattle behavior as well as farmer management interventions for crops, livestock, and woodland grazing area. [9] In addition to these interview notes (which took place between the late 1980s and mid-2010s), several of our coauthors are themselves successful farmers in Mazvihwa over different time periods collectively accounting for the 1980s through the late 2010s. Between interviews and accounts from our coauthors, we constructed rules in the agent-based model that corresponded to the behaviors of each system component (i.e. cows, crops, woodland). See below regarding the calibration and validation of these model rules.

Rainfall data from the Zimbabwean government

The Muonde team obtained monthly rainfall data from the Zimbabwean Department of Meteorology collected each year from 1950 through 2012 via rain gauges at three nearby locations: Zvishavane (20°18′37.52″S, 30°3′35.81″E), Chivi (20°18′52.73″S, 30°30′30.63″E), and Mberengwa (20°28′39.74″S, 29°54′27.14″E). The rainfall data are included in the model code, available on ComSES.net. We combine Muonde's crop yield data in kg/ha with the rainfall time-series to obtain a growth rate as a function of rainfall.

Parameters drawn from literature

In addition to the community-based field data and governmental rainfall data, we use values for some model parameters derived from the literature on African agriculture and pastoralism, where possible focusing on southern Africa and Zimbabwe in particular. These include metabolic rates for livestock [3, 10], energy density of various kinds of biomass in the system [4–7] and mass ranges for livestock [1, 2]. To decide how much additional variation in rainfall to introduce into different rainfall scenarios, we referred to literature on downscaling of global climate models to southern Africa [11, 12]. We also combine Muonde's field measurements on woodland growth with literature on woody [8] and herbaceous [13–16] production in southern African savannahs (and Mazvihwa in particular [17]) to arrive at a woodland growth rate for average rainfall. Details of these calculations appear in the following section.

Calculations and processes incorporated in NetLogo model

In this section we describe the details of the model. We begin with the biomass and energy accounting between trophic levels, including cow basal metabolism and the energy densities of different kinds of biomass, as well as the conversion efficiencies between them. We then describe cow reproduction and our simple two-stage population model for cows. We review the data and calculations behind biomass growth in crops and woodlands growth as well as their dependence on rainfall. We describe the feedbacks between the entities, specifically ploughing, fencing, and consumption of plant biomass by cows. We then detail different scenarios for rainfall variation, including some which generate rainfall time-series with more variation than has been historically observed. We describe the farmer management interventions we include in our model interface, and finally outline our choices for model initialization as well as stopping criteria based on biologically minimal sustainability characteristics of the model system.

The three explicit entities (*zvinhu zvitatu zvinonyatsoonekwa muraramiro wazvzvinhu zvitatu zvinonyatsoonekwa muraramiro wazvoo*) in this model are cows (agents, *zvinokwanisa kufamba kubva pane imwe nzvimbo kuenda pane imwe*), crops (patches), and woodland (patches, *zvinoramba zviri pazviri*). Note that by 'cow' we mean both male and female animals (*mombe*) and they are meant to represent livestock in general; by 'crops' we mean arable farming land area (regardless of the type of crop); and 'woodland' we mean all grazing land area. See the Overview, Design, and Details document (ODD, [18, 19]) on CoMSES.net for the details of variables that are tracked on a per-entity basis. [20]

Energy densities of biomass, conversion efficiencies, and cow metabolism

We account for biomass exchange between primary producers (crops and woodlands) and consumers (cows) by tracking an energy pool for each cow which reflects metabolic and working costs as well as the energy gains from consumption of plant matter. We then use energy densities for each of these types of biomass, and efficiencies in building new tissue or burning reserves when cows aren't able to eat, in order to convert that energy surplus or deficit to a mass gain or loss. Below, we review our logic behind the parameters for energy densities of cow, crop, and woodland biomass, for cow metabolism, and for efficiencies in burning or building cow tissue.

The energy density of cow varies from 1210-3320 kcal/kg depending on its body fat percentage (3-30%), based on the energy density in ground beef. [5] The cows in Mudhomori are lean, so we choose a 5% fat content, and a corresponding value of 1360 kcal/kg. Though this is a calculation from a U.S. agency, these statistics include meat with very low body fat. Therefore we assume that, for the purposes of our model, despite the differences between African and United States contexts, the fat content is the dominant factor in energy density despite potentially large geographic differences in breeds of cattle and their husbandry.

We use the following logic for the values of metabolizable energy in plant biomass (*chikafu chichashandiswa mumuviri wemombe kuipa simba*). For woodland browse, we compare with the metabolizable energy density of hay supplemented with nutrients from a study in Ethiopia, which was approximately 1931 kcal/kg. [3] The nitrogen content of the Ethiopian feed was 1.32% by volume; using a standard calculation for conversion from nitrogen to protein, [21] this is approximately 8.25% protein by volume. Compare with estimates for mopane and acacia browse: in Botswana, crude protein for *A. tortilis* dry leaves was 10.2%, mopane bark was 4.62%, and mopane old leaves was

13.4%, [22] which is consistent with values of crude protein for mopane leaves (9.2-13.9%), twigs (4.2-5.0%), and pods (8.6-15.9%) in a study combining data from Zimbabwe, South Africa and Botswana. [23] Because protein is perhaps the key nutrient in assessing the energetic quality of feed, we feel comfortable using the Ethiopian study's estimates of metabolizable energy for browse. [3]

Crop energy density is higher than browse: using the assumption for maize that grain is 42% of the plant by dry weight [6] and values given by Ohio State University Extension (maize stover, the non-grain portion of the plant, has a metabolizable energy of 0.79 Mcal/lb, and grain has a metabolizable energy of 1.42 Mcal/lb [7]), we calculate that a maize plant has a metabolizable energy of 2325 kcal/kg. Note that because these are already estimates of metabolizable energy (rather than gross energy), assimilation efficiency (i.e. how much material passes through a cow undigested) has already been taken into account in the method of measurement.

For cow metabolism, we use 'maintenance energy,' which includes basal metabolic functions, "thermoregulation, gut function, loss of energy in urine, and modest work for feeding and drinking." [10]. Values for maintenance energy rates vary susbtantially in the literature, for example 44 kcal/kg/day in [10] or, for our animals' weight range, from 720-2100 kcal/kg/day in [3]. We use an intermediate value, 980 kcal/kg/day. Our resulting maintenance energy loss (Δ_{E-}) multiplies this maintenance cost in kcal/kg (e_{maint}) by the current mass of the cow (m_{cow}):

$$\Delta_{E-} = e_{maint} m_{cow} \tag{1}$$

Regarding efficiencies in building new tissue or burning existing tissue, when the cow builds protein and fat from available metabolic energy, there is a production efficiency loss based on metabolic pathways. For protein the efficiency is 0.48 and for fat it is 0.71. [4] Because we assume a fat percentage of 5%, we weight these numbers accordingly and obtain 0.49 for the production efficiency (η_p) of building new protein and fat when a cow consumes excess calories. If a cow has not consumed enough to compensate for its maintenance energy, it loses body mass and liberates energy to address the deficit. This conversion has its own inefficiencies, however (from Johnson et al., protein degradation 0.90 and fat catabolism 0.95), which we weight in the same way as the production efficiency, to get 0.9025 for 'catabolism efficiency' (η_c) [4]. The mass change of a cow is therefore the following:

$$\Delta_m = (\Delta_{E+} - \Delta_{E-})\eta / e_{cow} \tag{2}$$

where Δ_m is mass gain in kg, Δ_{E+} is the energy gain in kcal from consuming plants and Δ_{E-} is the energy loss from metabolism and working, and e_{cow} is the energy density of cow in kcal/kg. If Δ_m is negative, η is η_c, and if Δ_m is positive, η is η_p. Note that in each time step, we check if a cow has lost enough mass to go below the minimum cow mass, in which case, the cow dies.

Cow reproduction

We adopt a simple two-stage population model of cows and calves in order to keep account of their different weight ranges (for simplicity, we do not distinguish males from females but give all adult cows a constant probability of reproducing). According to farmers, 50-60% of livestock are fertile females achieving 80% pregnancy in high rainfall years down to 50% in poor years and few in drought years, or a range from 0.25 to 0.48 probability of reproduction; in the model we use the lower estimate because most of our model years are not 'high rainfall years.'

In order to calculate a per-model-step probability of reproduction: if there is a 25% chance the cow reproduced this year (P_r), this means that the probability of not

reproducing all year (P_{nr}) is $1 - P_r$, or 0.75. This is the multiplicative result of not reproducing during each model time step throughout the whole year (p_{nr}): $0.75 = (p_{nr})^{(t_{yr})}$, where t_{yr} is the number of model time steps per year. The probability of not reproducing in a given model time step is then $0.75^{1/t_{yr}}$, and the probability of actually having reproduced in that time is the complement: $1–0.75^{1/t_{yr}}$.

For any cow with enough mass available to reproduce without reducing its mass below the minimum, we draw a random number from 0 to 1 and if it is less than p_{nr}, the cow splits into two cows, one with the minimum calf weight [2] and the other with the mass of the original cow (less the weight of the calf). This means that while we have nominally established a 25% reproduction rate per year (which, annually compounded over 60 years becomes 29%), in practice the rate may not match because not all cows are available to reproduce. At every model time step, we check the mass of each calf and when it is above the minimum mass for an adult cow plus a day's worth of maintenance energy, we reclassify a calf as an adult cow which is able to work and reproduce.

Annual crop and woodland growth and dependence on rainfall

We estimate crop growth with rainfall directly from community-based field data and Zimbabwean governmental rainfall records (Fig 1): fitting a linear function, we get a y-intercept of -203 kg/ha , a slope of 0.873 kg/ha/mm/year, and an x-intercept of 232 mm.

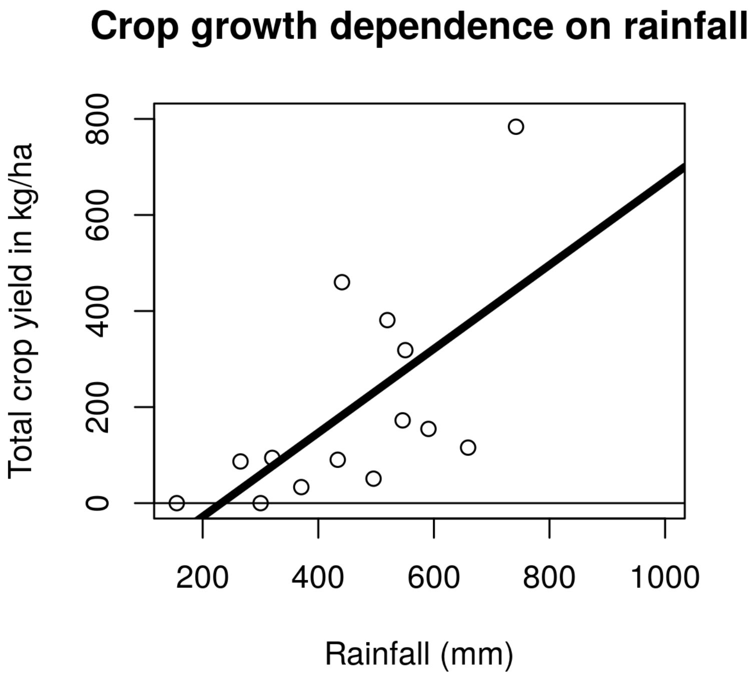

Fig 1. Crop yield as a function of rainfall. The linear fit is shown in the heavy dark line, with slope coefficient 0.873 (p=0.0132) kg/ha/mm and y-intercept -203 (p=0.188) kg/ha. Though the intercept is not significant, we know that crops will not grow without at least some rainfall so we make use of it in our model as a reasonable guess. In the model we refer to the zero-rainfall (x) intercept, which is the zero-crop (y) intercept divided by the slope, giving us 232 mm. (Figure from [20])

For the growth rate of woodland, we have community-based data on growth of acacia and mopane trees, and rely on literature review for herbaceous growth estimates as well as for comparison with our field-based measurements of woody growth. Livestock primarily consume herbaceous biomass, but also some woody biomass via browsing; likewise, herbaceous biomass cannot be used for fencing. We choose to simplify our model and pool all woodland biomass. To arrive at a total woodland biomass growth rate, however, we estimate herbaceous and woody primary production separately and then pool them.

From literature, in semi-arid savannahs of the type found in Mudhomori, but under lower livestock densities, the total productivity was on the order of 3000kg/ha/year, with nearly two thirds of this being woody biomass. [8] The primary production in the herbaceous layer of savannah grasslands in Southern Africa at around 500 mm annual rainfall has been found to be around 1200-1400kg/ha/year [13, 14, 16]; but these estimates were not made under the kind of heavy grazing regime observed in Mudhomori. [15] Long term heavy grazing and compaction on heavy clay soils like those in Mudhomori appears to reduce primary productivity, perhaps by as much as 50%, with most subsequent herbaceous production occurring in drainage lines and similar sites where water and nutrients then accumulate ("key resources" [17]). Therefore, we might estimate usable herbaceous layer primary production in Mudhomori's grazing areas at unlikely to be more than around 600-700kg/ha/year. For the model, we use 600 kg/ha/year.

From field measurements in Mazvihwa, *C. mopane* (*zita rinodaidzwa naro Mupani ku 'Science'*) has been recorded to grow at about 1000 kg/ha/year (at the coppicing stage at which most of this woodland now is) while *Acacia spp.* grew as much as 4000 kg/ha/year. In Mudhomori, approximately 80% of the woodland grazing area supports trees, and in 2013 the relative proportions of *Acacia* and *C. mopane* woodland was 80:20, so a weighted average gives approximately 2720 kg/ha/year ((4000*0.80+1000*0.20)*0.80). Therefore between herbaceous and woody biomass, 3320 kg are produced per year (2720+600); likely our number is higher than Rutherford's due to the coppicing (*kutema miti*) of the mopane woodland.

In this semi-arid system, and on the heavy clay soils found in Mudhomori, we assume no growth at zero rainfall and a linear relationship between rainfall and woodland growth. For average rainfall, which is approximately 530 mm, this results in a growth accumulation of approximately 6.26 kg per year per additional mm of rain. The linear relationship is supported by Rutherford's data, and is what the field team and local communities observe for this soil and vegetation type (mopane- and acacia-dominated woodland).

Feedbacks between entities: ploughing, fencing, and consumption

We model several feedbacks between cows, crops, and woodland, including the following: crops depend on cows through ploughing, crops depend on woodland for fencing material, and cows depend on woodland or crops for food intake.

In our model, crop patches do not grow if there are no cows with enough body mass to expend the energy to plow them. We use a value of e_{work} (the energy needed per area per time to plough a field) from a study in Ethiopia in which cattle expend 3.85 MJ/hr to plough a hectare of crop, [3] which we convert to 920 kcal/hr. Farmers in Mudhomori report that it takes four cows about two hours to plough a hectare, giving 8 cow-hours to plough one hectare (h_{work}). While farming conditions in Ethiopia are quite different from Mudhomori, we assume that metabolic cost is similar for the purposes of our model. We calculate the energy loss due to working by multiplying the

two: $E_{work} = e_{work}h_{work}$. When a cow ploughs a crop patch, E_{work} is added to Δ_{E-} in Eq (2) along with the maintenance energy required for that time step. Cows are proxies for each other in our model, distinguishable from each other only by body mass and energy available, so we select a single cow with enough energy to plough the entire patch and deduct the cost of the ploughing in a single model time step. In reality, this ploughing would require multiple days and/or multiple cows.

Crops must be fenced to keep livestock from eating them and reducing the harvest. Typically materials are cut from the woodland in order to build these fences, and termites then consume the fences over time, necessitating replacement. In our model, we deduct biomass from a woodland patch to protect a crop patch that has no fence. See Table 1 for the amount of biomass required and frequency of replacement (we assume a linear decay rate) and Appendix 2 for details on fence variable updating and tracking available woodland patches.

When cows try to find a nearby patch to eat, they consider the four patches directly above, below, and to the left and right (*nzvimbo dzekufurira dziri pedyo*; these are known as their rook-neighborhood or "Manhattan" neighbors; neighbors4 in NetLogo) as well as their current patch (also note that cows can share patches). They will try to move to whichever patch has the highest biomass, even if it is a crop patch with a fence. The cow's probability of breaking through the fence and getting into the crops depends on both how hungry the cow is (their satiety value, s_{cow}, ranging from 0 = starving to 1 = fully sated) and how intact the fence is (f_{qual}, ranging between 0 = not present and 1 = impenetrable). We combine the two in a linear model with respect to the two variables, discounted by the number of model steps per day (t_{day}) because a cow can try multiple times in a day to get into a particular crop patch:

$$s_{cow} = \frac{m_{cow} - m_{min}}{m_{max} - m_{min}} \tag{3}$$

$$f_{eff} = [1 - (1 - s_{cow})(1 - f_{qual})]^{(1/t_{day})} \tag{4}$$

where m_{cow} is a cow's body mass in kg, m_{min} is the minimum cow mass in kg (for cows or calves, as appropriate), m_{max} is the maximum cow mass in kg, and f_{eff} is fence effectiveness. If a random number between 0 and 1 is greater than fence effectiveness, then the cow breaks through and moves to the crop patch. If not, the cow stays on the patch it started on.

Once the cow has moved (or tried and failed to move), it may then consume biomass on its current patch. Regardless of its own body-mass, it will either eat enough to maintain the mass of a maximum-mass cow for one model time step, or eat whatever is on the patch (whichever is smaller). This means that a low-weight cow can build up its body mass, unless there is not enough woodland biomass. We reduce the patch's biomass appropriately and adjust the cow's current metabolic energy pool for the model time step accordingly, as follows:

$$\Delta_{E+} = m_{feed}e_{feed} \tag{5}$$

where Δ_{E+} is a cow's energy gain in kcal from consuming crops or woodland material (see Eq 2), m_{feed} is the mass of crops or woodland browse consumed in kg, and e_{feed} is the energy density in kcal/kg of either woodland browse (e_{wood}) or crops (e_{crop}). If the cow has successfully eaten crop biomass, we track the accumulated crop loss for validation against field measurements. After a cow has eaten, we check that their mass is above the minimum; if it is not, they die.

Rainfall scenarios

We use historical annual rainfall records to represent realistic water limitations on the system. For validation, we compare independent field data to outputs of models using

these historical rainfall scenarios. However, climate models predict increasing rainfall variation for Zimbabwe due to climate change, so we are also interested in exploring how the system behaves under increased variation in rainfall. [24] We justify our choice for the additional amount of variation based on downscaled climate models for southern Africa, which predict rainfall variation on the order of 146-292 mm/yr [12], where our historical standard deviation in rainfall is approximately 178 mm/yr. These models therefore predict 0.81 to 1.64 times our historical variation. Another study shows predicted rainfall anomalies in units of standard deviation with magnitudes of about 1.5 by the year 2100. [11] We therefore sought a relatively simple way to simulate increased year-to-year variation in rainfall with 1.5 times the variation in the historical rainfall data. Ultimately, we have six different rainfall scenarios, including both baseline scenarios (‘constant,’ ‘historical,’ ‘random,’ and ‘statistical-random’) and increased rainfall variation scenarios (‘extreme’ and ‘statistical-extreme’). The details of the scenarios are as follows:

1. ‘constant’: We use the mean value of rainfall, to compare all models with rainfall variation against a baseline.
2. ‘historical’: We use the historical rainfall time series as-is.
3. ‘random’: We use historical values but randomly resample them with replacement (a non-parametric bootstrapping approach).
4. ‘statistical-random’: We use the mean and standard deviation of the historical distribution to select rainfall values from a normal distribution (a parametric bootstrapping approach). The normal distribution is a reasonable choice to represent this particular time-series and is available within NetLogo.
5. ‘extreme’: We use the ‘extreme’ values of the existing historical data (only the upper and lower quartiles). This results in a similar mean to the historical data, and a standard deviation approximately 1.36 times the historical data. It has the property of still using rainfall values within the range of the historical data.
6. ‘statistical-extreme’: We use the mean of the historical data and 1.5 times the standard deviation of the historical data to draw rainfall values from a normal distribution.

The ‘random’ and ‘statistical-random’ scenarios allow us to have a sense of how important the particular ordering of historical rainfall was in the system’s outcomes, and allow nonparametric and parametric bootstrapping of historical results, respectively. The ‘extreme’ and ‘statistical-extreme’ scenarios are two different (nonparametric and parametric) ways to generate greater variation in rainfall than was historically observed. Note that in both cases in which we draw the rainfall from a normal distribution (‘statistical-random’ and ‘statistical-extreme’), we truncate the distribution at zero (-2.98σ and -1.99σ respectively), avoiding negative values for rainfall. The crop and woodland growth rates change annually in the model depending on these annual rainfall values.

Farmer management interventions

Farmers decisions are represented by the parameter choices in the NetLogo interface, through which they can make a variety of choices which affect the system (Table 6). In this sense, they are represented by NetLogo’s ‘observer.’ Farmers are also represented by adaptive decisions during the model run: they move cows based on patch quality and subsidize the cows when rainfall is too low, and also react to a crop patch having zero

biomass by seeking a cow to plough the patch. The farmer management decisions represented in the interface made at the beginning of a model run are then static, however. Farmers determine the proportion of crops and their spatial configuration (via a variable called 'clumpiness') and what percentage of crops or woodland grow faster than the rest (through farming innovations or woodland restoration projects). They determine how many times per day a cow is moved to a better grazing location, whether cows should be subsidized in low-rainfall years and if so, in what way: by transportation out of the village or by augmentation of their diet with supplemental feed, and what proportion of the cows to subsidize. Farmers also determine whether fences should be 'invincible' (meaning, stone walls instead of brush fencing) and how long to store crops. We review these management interventions below.

Table 6. Management-related variables (set through model interface). NetLogo variable names given in parentheses in first column; validation ranges given in parentheses in second column.

**Name**	**Units, Values & Data Source**	**Explanation**
Percentage land-use dedicated to crops<br>(proportion-crops)	1-99% ‡<br>(40-65%)	How much land is put into agricultural production?
Moran's I<br>(clumpiness)	-0.6-0.95;<br>'clumpiness' values = 0, 0.07, 0.22, 0.54, 0.85, 0.95, 1 †<br>(all values)	Represents the fraction of crop patches intentionally placed adjacent to other crops. (*minda yakabatana zvakadii / mamiriro akaita minda*)
"Move Cows"<br>(times-per-day-farmers-move-cows)	0 or 1 times/day ‡<br>(1 time/day)	How often per day do farmers move cows to better grazing? 0 means never.
"Stone Walls"<br>(invincible-fences)	True/false ‡<br>(False)	Stone walls ('invincible fences,' *ruzhowa rwusingapindike nemombe*) do not require woodland biomass and animals can't break through them.
"Subsidize Cows"<br>(subsidy)<br>(proportion-cows-to-save)	None, 'feed', or 'transport'<br>0.7, 1.0 ‡<br>('feed,' 'transport,' 0.7)	In a bad year, farmers can choose to buy supplemental feed or to transport cattle out of the village. (*kubatsira mombe nechimwe chikafu / nekuchinja nzvimbo*)<br>If subsidizing, what proportion of cows are subsidized?
"Preserve Forest"<br>(key-resources)	0%, 10% [17],‡<br>(10%)	What proportion of the woodland grows faster? (*nzvimbo dzakakosha mumasango dzinogona kuraramisa mombe*)
"Crop Innovations"<br>(muonde-projects)	0%, 10% ‡<br>(0%)	What proportion of the crops grow faster due to the Muonde Trust's projects to increase drought-resistant crops and water harvesting techniques?
"Store Grain"<br>(how-long-to-store-grain)	0 or 3 years ‡<br>(3 years)	How many years are farmers able to store harvest surplus due to bumper crop harvests in high rainfall years?

†The relationship between clumpiness and Moran's I is nonlinear, so based on an early model run we used a nonlinear estimation method to obtain a relatively even selection of Moran's I values with respect to clumpiness values, and in the BehaviorSpace selected only a few clumpiness values.
‡From community-based data collected by the Muonde research team.

Farmers over the years have grouped crops together and also scattered them throughout the woodland, so we simulate a variety of spatial configurations.

Unfortunately, the ‘clumpiness’ variable in the model interface is useful in creating the landscape configuration but not in measuring it. Therefore, we calculate two spatial autocorrelation metrics and two landscape ecology metrics to represent the spatial configuration. The two measures of spatial autocorrelation are Moran’s I [25] and Geary’s C [26]. For the weighting factors in these indices, we include the rook neighbors, all weighted equally. In addition to these measures, we calculate the total perimeter of crops and the average landscape patch size of the crop class, as defined in FRAGSTATS [27]. Note that we distinguish between ‘landscape patches’ and patches or cells in the Netlogo sense; here we refer to ‘landscape patches’ in the sense of landscape ecology: areas which are relatively homogeneous and differ from their surroundings, often classified as areas of different cover types. Empirically, our models give a Moran’s I from -0.6 to 0.95, a Geary’s C from 0.05 to 1.6, a crop perimeter from 1,176 m to 190,276 m, and an average contiguous landscape patch size for crops from 0.24 ha to 599 ha.

Some parts of the woodland grazing area grow much faster, even in low-rainfall years, than other parts. These areas can be sacred forests (*rambotemwa*), drainage ditches, contour ridges, and streams. The herbaceous, non-woody (*wezvusiri ‘woody’*) component of these “key resources” in the woodland grows up to ten times faster than other parts of the woodland, [17] or up to four times faster when woody growth is included in the calculation. In the 1980s, researchers estimated that up to 10-25 percent of the woodland area fell into this category; community observations suggest that the current amount is 10 percent or lower so we test 0% and 10%.

Similarly, some crop patches grow faster than others due to investments in agroecological innovation on the part of the Muonde Trust. These innovations include water harvesting techniques such as dead-level contours with paired ditches, water retention and infiltration basins (‘Phiri pits’), and planting of drought-resistant crop types and varieties (e.g. millets, sorghum). Either or both of these types of innovations could allow Muonde crop patches to grow faster than others. Because we do not yet have comprehensive empirical evidence of how much faster they grow, we use a factor of 3 times faster. We test scenarios in which 0% and 10% of crop patches have these innovations.

One innovation of the Muonde Trust is to build dry stone walls (built with interlocking stones but without mortar) rather than brush fences. These walls mean that livestock cannot break through no matter how hungry they are, and they also do not require woodland biomass to constantly repair due to termite damage. In the model, if farmers are using stone walls, we simply skip any fence updating processes and assume the fence is fully intact. Note, however, that if a cow wants to try to move to a crop patch with stone walls, the cow will still try but always fail to break through.

In Mudhomori, farmers can move cows to better grazing areas, typically multiple times a day. In the model, we test a situation where farmers move cows to the highest-biomass patches in the woodland once a day (as far as necessary), versus cows only moving on their own (one patch per model time step). In addition to this, farmers in Mudhomori are able to subsidize their cows during poor-rainfall years, either by feeding their cows supplementary feed or transporting them to a grazing area outside their village. In our model, if rainfall is less than 400 mm, the user can choose a proportion of cows to subsidize either by feeding or transporting them. Transporting means that farmers herded their cows to a different grazing area outside the village. This externalizes the cost of feeding the cow as it grazes in a different system, but this was a valid way in the past 60 years that farmers have kept their cattle alive. Alternatively, in the model, farmers can buy supplementary feed and apply it to subsidized cows. We assume an energy density for supplemental feed equivalent to that of crop, because farmers are likely to purchase feed which is higher-quality than the

woodland browse. We add the energy gain associated with eating the supplemental feed $m_{max}e_{maint}$ to Δ_{E+} in Eq (2) and add the cost of the feed to the subsidy accounting: $c_{crop}m_{max}e_{maint}/e_{crop}$, where c_{crop} is the cost of supplemental feed in US Dollars per kg of feed.

For some crops (such as small grains like sorghum and especially bulrush and finger millets, as opposed to maize/corn) harvests can be stored from year to year, up to three years (depending on quality of storage bins and storability of crop type and variety). This means that if there is a bumper harvest in one year, it may be possible to carry over the surplus into the next, potentially low-rainfall, year. In the model, we included three-year storage, averaging harvests over the previous three years, or no storage.

Model initialization

The goal of initialization for our model was to avoid including transient behavior dependent on initial conditions. We implemented this by randomizing some variables for each patch (fence quality for crops and biomass amount for woodlands), and setting others close to an estimated equilibrium value (initial number of cows: see below for details on calculating the woodland's carrying capacity), and then running the model for some time with constant rainfall to let transient behavior (associated with these random starts and the particular spatial configuration) fade. Once the model had a chance to reach equilibrium, we then began monitoring variables and (in some simulations) varying rainfall. After observing a variety of model runs, we determined that five model years was sufficient to allow transient behaviors to fade. (See Appendix 2 and ODD [20] for technical details of how the cows', crops', and woodlands' entity variables are set when the model is initialized.)

To set the initial number of cows as close to equilibrium (under constant rainfall) as possible, we calculated the potential carrying capacity of the woodland. This calculation was based on the average of the maximum and minimum weights of cows (m_{max} and m_{min}, Table 1): 179.5 kg. We summed the total biomass of the woodland generated per unit time, based on proportion crops, Netlogo patch-size, and adjusted for a potential proportion of the woodlands which grow four times as fast, or three times more additional patches' worth of growth ('key-resources,' see above and [17]). We then converted that biomass to cow biomass using the metabolic models given above, then divided that mass by the ongoing needs of a reference cow, in order to get the potential number of cows supported by that amount of woodland and key resources. The ongoing needs were those associated with metabolizing and moving, not with reproduction or ploughing/working. Note that cow carrying capacity could be larger with subsidies, when cows are able to break through fences and eat crops, with higher-than-average rainfall, and when the initial values for the woodland growth-states happen to be higher (i.e. before the transient behavior dissipates). Empirical testing with the model indicated that this calculation did achieve a carrying capacity which started very close to a stable state (when rainfall is not varied). The calculation was as follows:

$$E_{tot} = P_{wood}A_{Mud}(1 + 3P_{keyres})g_{wood}R_{avg}e_{browse} \tag{6}$$

$$E_{cow} = m_{cow}e_{maint} \tag{7}$$

$$C = E_{tot}/E_{cow} \tag{8}$$

where E_{tot} was the total energy available from woodland biomass in kcal/day, P_{wood} was the proportion of the land area set aside as woodland grazing area, A_{Mud} was the area of Mudhomori village in hectares, P_{keyres} was the proportion of woodland that grows faster ("key resources"), g_{wood} was the woodland growth rate in kg/ha/day/mm, R_{avg} was the average rainfall in mm, e_{browse} was the metabolizable energy density of browse in kcal/kg of browse, E_{cow} was the energy cost of a reference cow in kcal/day,

m_{cow} was the mass of an average cow in kg (calculated from the average of max and min cow mass), e_{maint} was the cow maintenance energy cost in kcal/day/kg of cow, and C was the carrying capacity in number of cows. (Note that to set the initial number of livestock agents, we rounded to the nearest integer.) For the range of input variables (1-99% crops and 0-10% key resources), we had a range of initial cow numbers from 1-77. This value may be somewhat lower than necessary for some models in which cows were subsidized or allowed to consume crops by breaking through fences; however, this calculation was a reasonable starting place before allowing the model to run for five model years.

Sustainability thresholds (stopping criteria)

Because our interest was in studying complex system sustainability, we created an outcome variable called 'sustainable' which was 1 if all three components (cows, crops, and woodland) met a minimum quantity for all 60 model years of the simulation (and 0 otherwise). We sought biologically minimal thresholds for each component in order to give the greatest number of successful runs with which to investigate the validation of the overall model and the relative sensitivity of results to input parameters. The logic behind the idea of minimum thresholds is that farmers would not be able to buy cows, seed, or nursery-raised woodland trees, and instead would need at least so many cows, so much seed, or so much woodland biomass for these components to maintain themselves. We recognize that in reality these thresholds would represent an untenably optimistic definition of 'sustainability,' but they are adequate for the purpose of exploring model behavior. We describe each of the minimum thresholds below.

For a biologically minimal number of cows, we used the simple requirement that there must be two cows in order for new cows to be produced. Though we did not model the sex of cattle or the metabolic cost of reproduction on female cattle (only the loss of the mass of the calf), the biological minimum should still be at least two cows.

For harvest, we used the requirement that the harvest must at least be large enough to generate enough seed to replant the fields. One farmer on our team (E. Mhike Hove) reported that it takes 10 kg/ha to plant maize, and more for small grains (sorghum, millet). Another long-term outsider researcher on the team (K.B. Wilson) reported 2 kg/ha. Internet sources from elsewhere in Africa (Uganda) suggested that the number should be higher, e.g. 25 kg/ha [28]. Because E. Mhike Hove is currently a successful farmer in Mazvihwa, and his estimate was intermediate, we used 10 kg/ha.

Our proportion-crops in the model can range from 1% to 97%, so our estimate for this minimum depended on the proportion-crops. Because even one crop NetLogo-patch (in our model, 0.24 ha in size) being seeded would allow us to have some crops (even if we had hoped to seed all 97% of the landscape), we use the minimum (1% of the village area). Mudhomori village is approximately 600 ha in size, therefore 10 kg/ha * 600 ha * 1% is 60 kg, or 0.06 metric tons.

Finally, for minimal biomass in the woodland grazing area, we used the logic that for trees to re-grow in the woodland, a seed source would be necessary, i.e. an adult tree of reproductive size. K. B. Wilson suggested that the mass of an adult acacia tree is 50-400 kg, and 200-500 kg for an adult mopane tree. To triangulate this estimate, we reviewed the allometric literature for southern African tree species, especially acacia and mopane. Ultimately we chose mopane as our benchmark for the minimum threshold, due to its cultural and agroforestry importance to the community, and because more allometric data was available.

We used an equation relating diameter and biomass for *C. mopane* in Kruger National Park, South Africa (see first line on the left side of Table 1 in [29]), and an equation relating height and diameter (also for *C. mopane* in Kruger National Park, South Africa) as follows (see Table B1, *C. mopane* inside exclosures, in the linear range

of the function in [30]):

$$\ln B = -2.77 + 2.49 * \ln D \tag{9}$$
$$\ln H = 4.51 + 0.74 * \ln D \tag{10}$$

where B is tree biomass in kg, D was tree diameter in centimeters, and H was height in centimeters. Rearranging the equations and combining them, we obtained a relationship between biomass and height:

$$B = \frac{H^{(2.49/0.74)}}{e^{(2.77+4.51*2.49/0.74)}} \tag{11}$$

In the Luangwa Valley of Zambia, *C. mopane* fruits at 5 m (500 cm) tall [31]. At this height, the biomass would be 19.4 kg ($\sim$ 0.02 metric tons). For a tree 1000 cm tall, the biomass would be 200.0 kg (0.2 metric tons), which agrees with K.B. Wilson's judgment, so perhaps trees in Mazvihwa fruit at a much greater height than those in Zambia. We used the smaller of the two estimates, however, to allow as many models to succeed as possible in order to study a range of model behaviors.

Additional validation information

Regarding the subset of the model runs we used for validation, the actual proportion of crops has varied from 40% in the mid-1980s to 65% in recent years. Therefore we check model outputs based on this range. The degree to which crops have been clumped has varied, so we examine results for all of the 'clumpiness' values. Scoones [17] measured the area of woodland with "key resources" as 10% historically, so we use 10% key resources. Muonde projects are a recent innovation, so we check results for 0% Muonde projects. Stone walls are similarly a new innovation, so we check results for invincible fences = FALSE. We use rainfall-type = 'historical' because this is the actual rainfall time-series. Farmers do move their cows, so we use a value of 1, and animals were subsidized, so we check results for both subsidy = 'transport' as well as 'feed.' The research team reports that 70% of farmers used supplemental feed, so where we subsidized with feed, we look at results for 70% of the cows being subsidized, though when we compare the results of the model with the amount spent on subsidy, we omit the transported cows because our model only allowed one or the other. Farmers have been storing crops, so we look at results for three years of crop storage. We look at results for only models that ran all 60 years because the real system has in fact persisted.

Regarding the field data we used to compare with the model results from that subset (in the main paper): for cows we have from interview data that the total number of cattle in Mudhomori historically ranged from 5 to 200 (Table 4); and for harvest, we combine the average harvest quantity for our sample households in kg/ha (Table 3) with the size of Mudhomori village (600 ha) and the estimated proportion of the village that was used for growing crops (Table 2) in the years of those harvests to obtain a harvest for the whole village in each of these years (note that we use a linear fit to predict the proportion of the village used for cropping in years with no estimate). For cattle and harvest, we use percentage difference in mean and standard deviation to assess differences between yearly model values and yearly field data values. Based on our amount of field data, we were not expecting a high enough agreement to warrant a more sophisticated statistical test of distributional difference, for example a Kolmogorov-Smirnov test.

For the following three quantities, we only compare order of magnitude because the field data are single values or ranges. We compare with distributions of variables that

summarize a model run (for example, the minimum woodland during an entire run), rather than comparing yearly values from within each model run.

Minimum woodland biomass

We do not have historical estimates of the total amount of biomass in the woodlands; this is an active question of research for the Muonde team. The Muonde team estimates that there is currently about 1/20th the amount needed to re-make all of Mudhomori's current fences, so we use this as a benchmark (288 metric tons/20 = 14.425 metric tons).

Feed supplementation

The research team reports that 70% of farmers used supplemental feed, at an average of approximately 240 kg/household. There are 99 households in Mudhomori, listed in interviews and verified via Google Earth; for 99 households at 240 kg/year at $14.50/ 50 kg, this gives a total cost of 0.70*99*240*14.50/50 or approximately $4,800 per year.

Amount of crop eaten by animals breaking through fences

From interviews with farmers, estimates of the damage after animals had broken into fields indicated that three donkeys could eat 50% of 424 maize plants (out of 5500 plants, effectively 212 whole plants out of 5500) after 10-25 minutes (this is 3.9% of a farmer's crop as estimated by the farmer).

Using the standard that one donkey is 1/2 Tropical Livestock Unit and one cow is 2/3 Tropical Livestock Unit [32], 3 donkeys is approximately 2 and 1/4 cows. Therefore the consumption rate is 212/(2 1/4), or 94, plants per cow per 10-25 minutes.

Each ear of maize has 300-1000 kernels, weighing between 190-300 g/1000 kernels, usually with one ear per stalk, and the grain makes up 42% of the plant by dry weight, giving a weight for maize plants ranging between 1.53 to 20.2 kg per plant. [6] The crops eaten by the donkeys were reported to be only about 5% of an adult plant in size (corresponding to about 30 cm high).

This means that a single cow eating the crops in a similar fashion to the donkeys would consume between 0.86 and 11.4 kg of crop per hour. Note that we do not know how much more food the animal would eat if it were able to stay in the field for a longer time period (the donkeys were evicted from the field as soon as they were found); at some point the animal would no longer be hungry and would not continue to eat, and our cows are not modeled as consuming food in a panic as farmers attempt to remove them from the field.

References

1. Machila N, Fèvre EM, Maudlin I, Eisler MC. Farmer estimation of live bodyweight of cattle: Implications for veterinary drug dosing in East Africa. Preventive Veterinary Medicine. 2008;87(3):394–403.

2. Nicholson MJ. Calf growth, milk offtake and estimated lactation yields of Borana cattle in the Southern Rangelands of Ethiopia. No. 6 in Joint Ethiopian Pastoral Systems Study; 1983.

3. Astatke A, Reed JD, Butterworth MH. Effect of diet restriction on work performance and weight loss of local Zebu and Friesian X Boran crossbred oxen. ILCA Bulletin. 1986;23:11–14.

4. Johnson IR, France J, Thornley JHM, Bell MJ, Eckard RJ. A generic model of growth, energy metabolism, and body composition for cattle and sheep. Journal of Animal Science. 2012;90(13):4741–4751.

5. USDA. Ground Beef Calculator;. Available from: http://ndb.nal.usda.gov/ndb/beef/show.

6. Food and Agriculture Organization of the United Nations. Maize in human nutrition. vol. 25 of FAO Food and Nutrition Series; 1992. Available from: http://www.fao.org/docrep/t0395e/T0395E01.htm.

7. Eastridge M. Feeding Corn Stover to Ruminants. Buckeye Dairy News. 2007;9(3).

8. Rutherford MC. Primary production ecology in southern Africa. In: Werger MJA, editor. Biogeography and ecology of southern Africa. Dr W. Junk bv Publishers The Hague; 1978. p. 621–659.

9. Manson S, An L, Clarke KC, Heppenstall A, Koch J, Krzyzanowski B, et al. Methodological issues of spatial agent-based models. JASSS-THE JOURNAL OF ARTIFICIAL SOCIETIES AND SOCIAL SIMULATION. 2020;23(1).

10. Molden D. Water for food water for life: A comprehensive assessment of water management in agriculture. Routledge; 2013.

11. Shongwe ME, Van Oldenborgh GJ, Van Den Hurk B, De Boer B, Coelho CAS, Van Aalst MK. Projected changes in mean and extreme precipitation in Africa under global warming. Part I: Southern Africa. Journal of Climate. 2009;22(13):3819–3837.

12. Jury MR. Climate trends in southern Africa. South African Journal of Science. 2013;109(1-2):1–11.

13. Knapp R. Pflanzenarten-Zusammensetzung, Entwicklung und natürliche Produktivität der Weide-Vegetation in Trockengebieten in verschiedenen Klima-Bereichen der Erde. In: Knapp R, editor. Weide-Wirtschaft in Trockengebieten. Stuttgart: Gustav Fischer Verlag; 1965. p. 71–97.

14. Bourlière F, Hadley M. The ecology of tropical savannas. Annual Review of Ecology and Systematics. 1970;1:125–152.

15. Barnes DL. Defoliation effects on perennial grasses—continuing confusion. Proceedings of the Annual Congresses of the Grassland Society of Southern Africa. 1972;7(1):138–145.

16. Kelly RD. A comparative study of primary productivity under different kinds of land use in southeastern Rhodesia [PhD Thesis]. University of London; 1974.

17. Scoones I. Patch use by cattle in dryland Zimbabwe: farmer knowledge and ecological theory. Overseas Development Institute London; 1989. 28b.

18. Grimm V, Berger U, Bastiansen F, Eliassen S, Ginot V, Giske J, et al. A standard protocol for describing individual-based and agent-based models. Ecological Modelling. 2006;198:115–126.

19. Grimm V, Berger U, DeAngelis DL, Polhill JG, Giske J, Railsback SF. The ODD protocol: a review and first update. Ecological Modelling. 2010;221(23):2760–2768.

20. Eitzel MV, Neves KT, Solera J, Wilson KB, Mawere Ndlovu A, Fisher AC, et al.. Zimbabwe Agro-Pastoral Management Model (ZAPMM): Musimboti wevanhu, zvipfuo nezvirimwa; 2018. Available from: https://doi.org/10.25937/ta23-sn46.

21. Food and Agriculture Organization of the United Nations. Food energy - methods of analysis and conversion factors. vol. 77 of Fao Food And Nutrition; 2003. Available from: http://www.fao.org/tempref/docrep/fao/006/y5022e/y5022e00.pdf.

22. Dambe LM, Mogotsi K, Odubeng M, Kgosikoma OE. Nutritive value of some important indigenous livestock browse species in semi-arid mixed Mopane bushveld, Botswana. Livestock Research for rural development. 2015;27(10).

23. Makhado R, Potgieter JM, Luus-Powell W. Nutritional value of Colophospermum mopane as source of browse and its chemical defences against browsers: a review. Journal of Animal and Plant Science. 2016;26(3):569–576.

24. World Bank Group. Climate Change Knowledge Portal;. Available from: http://sdwebx.worldbank.org/climateportal/index.cfm?page=country_future_climate_down&ThisRegion=Africa&ThisCcode=ZWE.

25. Moran PA. Notes on continuous stochastic phenomena. Biometrika. 1950;37(1/2):17–23.

26. Geary RC. The contiguity ratio and statistical mapping. The incorporated statistician. 1954;5(3):115–146.

27. McGarigal K, Cushman SA, Ene E. FRAGSTATS v4: spatial pattern analysis program for categorical and continuous maps; 2012. Available from: http://www.umass.edu/landeco/research/fragstats/fragstats.html.

28. Adefaru L. Easy ways to plant maize for high yields; 2018. Available from: https://www.monitor.co.ug/Magazines/Farming/Easy-ways-plant-maize-for-high-yields/689860-4354292-px92jr/index.html.

29. Nickless A, Scholes RJ, Archibald S. A method for calculating the variance and confidence intervals for tree biomass estimates obtained from allometric equations. South African Journal of Science. 2011;107(5-6):86–95.

30. Moncrieff GR, Chamaillé-Jammes S, Higgins SI, O'Hara RB, Bond WJ. Tree allometries reflect a lifetime of herbivory in an African savanna. Ecology. 2011;92(12):2310–2315.

31. Caughley G. The elephant problem–an alternative hypothesis. African Journal of Ecology. 1976;14(4):265–283.

32. Le Houérou HN, Hoste CH. Rangeland production and annual rainfall relations in the Mediterranean Basin and in the African Sahelo Sudanian zone. Rangeland Ecology & Management/Journal of Range Management Archives. 1977;30(3):181–189.
