# Supplementary material for: Using mixed methods to construct and analyze a participatory agent-based model of a complex Zimbabwean agro-pastoral system
Source: PLoS One. 2020 Aug 21;15(8):e0237638. doi: 10.1371/journal.pone.0237638 (PMC7442250; doi:10.1371/journal.pone.0237638)
Supplement: S2 Appendix — Different model modes, details of software unit tests, profiling, and behavior tests, how we perturbed parameters for sensitivity analysis, how we tracked NetLogo patches and agents for better model updating efficiency, spatial and temporal scale restrictions of the model, details of creating crop spatial configurations and other initialization, notes about some of the updating procedures, and an outline of the order in which model functions (NetLogo procedures) are called. (PDF) [file pone.0237638.s002.pdf]

S2 Appendix: Details of technical implementation. Different model modes, details of software unit tests, profiling, and behavior tests, how we perturbed parameters for sensitivity analysis, how we tracked NetLogo patches and agents for better model updating efficiency, spatial and temporal scale restrictions of the model, details of creating crop spatial configurations and other initialization, notes about some of the updating proceedures, and an outline of the order in which model functions (NetLogo procedures) are called.

Model Modes ***(nzira dzinokwanisa kushandiswa nadzo ' model' iyi)***
We had three different 'model modes': 'Experiment' 'Demonstration' and 'SoftwareTest.' This is a user-chosen variable. Demonstration mode did not include burn-in (so a user does not need to sit and wait for burn-in to finish), and uses fixed values of the underlying (non-user-set) parameters. The initial value for rainfall is simply the mean rainfall, the first value in the historical timeseries, or the first randomly chosen value based on the climate variation model This was the mode to use to visually explore model behaviors; it also displayed the final values of the reporters in NetLogo's output area.

Experiment mode did not update the display (to save run time) and perturbed the underlying parameters by 5% in order to assist with sensitivity analysis, as well as having a five-model-year burn-in to allow transient behaviors associated with initial conditions to fade. "Burn-in" runs the model for five model years at the mean rainfall value for the chosen time-series (from one of three nearby locations: Zvishavane, Mberengwa, and Chivi, or their average) to allow the transient model behavior inherent in the spatial configuration and the random starts for fences and woodland growth-states to die off. Then the simulation begins recording monitor variables and varying rainfall. This mode was intended to be used in NetLogo's BehaviorSpace tool. SoftwareTests mode was for checking the basic functionality of the model procedures.

Profiling
We used Netlogo's profiling tool to assess which functions were taking the longest, in order to help reduce run times as much as possible. This was important for both community use of the model as a discussion tool (they could see the results of more different combinations in a shorter amount of time), and in saving compute time when running the sensitivity analysis (allowing us to run almost 500,000 simulations in just over 10,200 core-hours). The final version of the code does not include the profiling tool, but in earlier versions we used the tool by creating a button in the interface with the following code embedded:

```
setup                               ;; set up the model
set burn-in false                   ;; make sure we're measuring everything
reset-timer                         ;; start NetLogo's timer to zero
profiler:start                      ;; start profiling
repeat 10 * ticks-per-year [ go ]   ;; "go" runs everything
profiler:stop                       ;; stop profiling
print profiler:report               ;; view the results
profiler:reset                      ;; clear the data
print timer                         ;; in seconds
```

Behavior Testing ("Face Validation")
We also checked for a set of known behaviors when we made changes to the code (Ngo and See 2012). This was tracked using a spreadsheet (included in the supplemental information) which indicated input values to select in the model interface, and a checklist of expected behaviors and emergent properties. For example: 'invincible-fences' = TRUE means 'crop-eaten' = 0 (stone walls keep cows from eating crops) or 'rainfall-type'='constant' means "subsidy-used" = 0 (subsidy only happened in low rainfall years, so constant rainfall means it should never happen).

Software/unit testing
We created a 'model-mode' designed for testing the individual outcomes of each function as a software best practice, also referred to as 'unit testing.' Unit testing is not embedded in NetLogo's functionality, so in the 'setup' function, we checked for model-mode = "SoftwareTest" and ran a function which then called all the individual software tests ("run-software-tests"), which runs all the software tests and displays whether each test was passed (in the output area). In our BehaviorSpace sensitivity tests/parameter studies, we include one BehaviorSpace which simply runs all the tests, so that every set of model outputs also has a record that the tests were working.

We wrote standard functions used in unit tests ("assert", "assert-equal", "assert-float-equal") and then individual functions which tested the outcome of one of our model functions, for example setting up a known spatial configuration and testing the assertion that Moran's I and Geary's C calculations result in a known value (verified by manual calculation), or creating one cow and setting its mass to a value low enough to cause it to die during the "consume" function, and checking the assertion that the "count cows" is zero after "consume" is called. The unit tests were designed to be run any time a change is made to the code to make sure we avoided introducing unintended behavior or bugs.

The unit tests each test a single base-level outcome of our functions, and therefore take a large amount of space in the code. In software with built-in unit testing capability, this code would be displayed in another model tab, and perhaps NetLogo could in future iterations have this kind of functionality. This would encourage modelers to think in terms of small enough model outcomes to write robust unit tests and would make agent-based models as a method more robust and defensible, particularly because of their heavy use of programming (as opposed to, for example, largely statistical models using canned procedures).

Perturbation functions for sensitivity analysis
We considered using the standard errors of estimates from our crop and woodland growth models to constrain the sensitivity analysis. However, none of the estimates of parameters drawn from the literature had standard errors, so we chose to keep our sensitivity analysis methods consistent across variables, perturbing them by +/- 5%. We did vary 'Muonde efficiency' by +/- 10% because it was particularly poorly known; and 'woodland growth rate' by +/- 10% to encompass both our estimate and those from the literature.

Keeping track of available patches/agents
What the profiling revealed was that searching through all agents or patches was extremely time-intensive: making NetLogo's 'ask' loops and other searches of agents or patches more efficient

was absolutely critical to reduce model run time. Therefore, we kept account of the Netlogo patches and agents which were available for various uses (ploughing, fencing, reproducing, grazing), because not all agents or patches will be available and this reduced the searching time in NetLogo. These sub-sets of agents and patches were dynamically updated after events like ploughing, repairing fences, reproducing cows, and so on. This efficiency was important, because our model's complexity and varied data sources necessitated sensitivity testing of management and underlying variables – and that meant running many different versions of the model.

Spatial and temporal scales of model

Many of the model's parameters and processes have been made scale-invariant by multiplying or dividing by ticks per day or hectares per patch; however, several points should be noted. First, in our implementation, ticks-per-year must be an integer because we rely on the modulus operator to determine when the calendar year should change. Second, a world size of 50x50 means that each patch is approximately a quarter hectare, and 3 ticks per day means a tick is 8 hours. We get realistic results for these parameters. The temporal and spatial scales could be adjusted, but the user should be aware that the rate at which a cow moves across the landscape when grazing should remain realistic, as should the amount of time it takes to plough a field (though the energy is deducted all at once).

Creating spatial configurations using 'clumpiness' variable

Once the global variables are set, we make all patches 'woodland,' count the number of patches (world-size * world-size) and based on proportion crops, establish a desired number of woodland and crop patches. We use the 'clumpiness' variable to establish a number of crops that should be in clumps, with the rest of them scattered evenly throughout the woodland. (Clumpiness is therefore a proportion of the crops that should be intentionally placed in clumps. Note that some crop patches may end up clumped by random chance.)

We then make crop 'clumps' by first placing the 'non-clumped' crops somewhat evenly throughout the woodland, creating a chess-board-like pattern (making a woodland patch a crop patch if it has all woodland neighbors or failing that, if it has at least one woodland neighbor), and then by placing the 'clumped' crops (making a woodland patch a crop patch if it has all crop neighbors or failing that, if it has any crop neighbors). Which patches are crop and woodland, and what spatial configuration they are in, is randomly generated for each of our model runs.

This procedure results in a nonlinear relationship between 'clumpiness' and measures of spatial autocorrelation, but it is at least monotonic. For the large parameter sweeps, we chose several values of 'clumpiness' that would give a somewhat even distribution of spatial configurations as measured by our landscape ecology variables.

To calculate these measures of spatial configuration, we set the variables moran-numerator, geary-numerator, and moran-geary-denominator for each patch by asking it how its class (crop or woodland) compares to its neighbors and weighting appropriately for Moran's I and Geary's C (see code for details). We then calculate the global indices based on each patch's numerator and denominator. For total crop perimeter, we take the sum of how-many-fences over all crops-with-fences, multiplied by the length of the side of a patch based on world-size. For average

contiguous crop cluster size (average landscape patch size of crops), for each crop, grow a cluster with only crop neighbors, record the cluster size in a list, remove those crops from all available patches, choose a new crop patch and repeat the procedure until no crop patches remain. Then average the sizes of the crop clusters in the list.

Initializing cow agent variables
We calculate the carrying capacity of the current woodland configuration and create that many cows. We place each cow randomly on a woodland patch (cows can share patches). Initially set satiety to 0.51, is-subsidized? to FALSE, and is-calf? to FALSE. We set a cow's body-mass to be an average of the minimum and maximum cow mass, which we obtain from a study of a number of different ways to weigh cattle in Kenya (Machila et al. 2008). We use their weight ranges from the 'gold standard' weighing methods that use a calibrated scale: 63-296 kg for adult cows. A mean birth weight of calves in Ethiopia was found to be 18 kg (Nicholson 1983), so when we create new calves, we use this minimum mass, set 'is-calf?' to TRUE, and make the icon representing the calf half the size of an adult. Initially we set energy-this-tick to one tick's worth of maintenance energy.

Initializing crop and woodland patch variables
Biomass
We set standing-available-biomass (*huwandu hwezvinhu zvese zvinorarama panzvimbo*) to zero for crops and randomly between 0 and 5% of a year's growth for woodland (0 to 39.84 kg/patch). We choose between 0 and 5 % so that the livestock number and woodland biomass can equalize quickly during burn-in. We set it low because we will set cows at the carrying capacity of the woodland and therefore they should maintain their population as the woodland grows. We do not set it to zero because there is some stochasticity in the system and the additional amount is a buffer preventing unnecessary cow population crashes.

Fences
We create a set of crop patches with any Manhattan/rook neighbors (NetLogo's neighbors4) that are woodland as "crops-with-fences" and then for each crop with a fence, count how many woodland neighbors it has. We set all crops with fences to have a fence variable randomly between 0 and 1 (completely gone and completely intact). One innovation of the Muonde Trust is to build dry stone walls (built with interlocking stones but without mortar) rather than brush fences. These walls mean that livestock cannot break through no matter how hungry they are (how low their biomass is), and they also do not require woodland biomass to constantly repair due to termite damage. In this case, we set fence to 1 (completely intact).

Fence updating
To update the fences in the model, we first collect a list of woodland patches with enough biomass to build a fence. Then, for each of the crops which border woodlands, the fences get eaten by termites: reduce the `fence' variable by 2/3 divided by ticks-per-year. Then, for crop patches with fence = 0, repair fences: remove 5.77 kg/m * the length of the side of a Netlogo patch of biomass from an available woodland patch, one side at a time; repeat as necessary for all the sides of the crop patch that border a woodland patch. Update the "fence" variable appropriately: for example, if the crop had three sides bordering woodland and we were able to repair one of them, set `fence' to 1/3.

Harvesting crops
To harvest crops in the model, we first update annual crop trackers which include a running list of the last several years' harvests (number of years set by the user as how-long-to-store-grain). We drop the oldest element of the list, then sum all the standing available biomass of the crop patches, and add that sum to the list as the current year's harvest.

Farmers moving cows
Before moving any cows, we (globally) search all the woodland patches for a list of enough potential locations with the highest available biomass. This list will contain at maximum either all the woodland patches (at least as many cows as patches) or as many patches as there are cows. After a cow has moved and consumed, we update the list by removing the patch with the least biomass from the list of potential destinations.

Process overview and scheduling (***pfupiso yamashandiro uye marongerwe azvakaita***)
Here we give the details of which NetLogo procedures are called and in what order.

Function names in the actual code are underlined; conditionals and loops are *italicized*. Cows, woodlands, and crops are asynchronously (***munguva dzakasiyana***) updated (immediately and in random order) every tick, and all updates are per tick unless otherwise noted.

"Setup" (before the model runs):
set (initialize) global variables
make crops (and woodlands): make-crop-clumps: initialize spatial configuration
set (initialize) patch variables (crops and woodlands)
configure fences for crops
calculate-landscape-metrics
set-faster-growing patches
create-cows: calculate-initial-number-of-livestock and initialize state variables

"Go" (each tick/model step during model run):
update max/min; monitor variables for woodland, number of cows (*after burn-in is over*)
yearly: check-burn-in-and-and-update-year (both calendar year and years-gone)
yearly: check-timeseries-length; *after 60 years*, stop simulation
update-yearly-time-events
- *yearly*: check-cow-woodland-thresholds *if too little*, stop simulation (*after burn-in is over)*
- *yearly*: get-new-rainfall & set-new-growth-rates for patches accordingly
- *yearly*: set-subsidized-cows *if farmers are subsidizing cows and rainfall is low enough* (***kupa mombe chimwe chikafu***)

update-cows (*loop through agents*)
- *if farmers are moving cows*, globally find suitable patches anywhere within the grazing area
- *if some cows are being transported outside the village*, 'freeze' them; *otherwise:*
- move
  - *if farmers are moving cows*, every so many ticks get moved by a farmer
  - *otherwise* find a nearby patch to eat (try to get through a fence if necessary)

move-to new location
consume
eat what is on the patch, or enough to reach max body mass, adjust energy accordingly
reduce the available biomass of the patch
update-crop-woodland-eaten trackers
get hungrier
adjust energy pool of cow to deduct maintenance energy
convert cow energy pool to mass change and update satiety
subsidize with supplemental feed as necessary (and adjust energy/mass again)
check-calf-status and graduate calves to cows *if body-mass is high enough*
if body mass is too low, starve
update-globally-suitable-patches list
color hungry cows red and find available cows for ploughing/reproducing in next tick
reproduce cows
if a cow has enough body mass, based on a random number draw, give-birth
update-available-cows
update crops (*loop through crop patches*)
if no stone walls: update-fences: *(only loop through crops with fences)*
find available woodland patches for fencing
get eaten by termites
if any available woodland for fencing, repair-fences
if available biomass is zero and cows are available, plough:
grow crops and adjust energy of cow, update-available-cows for working/reproduction
otherwise, grow crops: update available crop biomass based on current rainfall-driven growth rates
update crop color/symbol to represent available biomass, condition of fences
yearly: harvest:
update-annual-crop-trackers for crop eaten and running list of harvests
reduce available biomass to zero
update-and-check-total-harvest; *if average harvest is inadequate*, stop simulation
update woodland available biomass (*loop through woodland patches*) grow and update symbology
advance-time-step: increment the model time step (tick), check to see if a calendar year has passed

When the model run ends (has met a stopping condition):
finish running and clean up:
Calculate actual reproductive rate (a per cow per tick variable)
Calculate amount of crop eaten per half hour (a per cow per tick variable)
Record the reason for termination (too little woodland, cows, or harvest, or 60 years has passed)

References

Machila N, Fèvre EM, Maudlin I, Eisler MC. Farmer estimation of live bodyweight of cattle: Implications for veterinary drug dosing in East Africa. Preventive Veterinary Medicine. 2008;87(3):394–403.

Ngo TA, See L. Calibration and Validation of Agent-Based Models of Land Cover Change. In: Heppenstall AJ, Crooks AT, See LM, Batty M, editors. Agent-Based Models of Geographical Systems. Dordrecht: Springer Netherlands; 2012. p. 181–197

Nicholson MJ. Calf growth, milk offtake and estimated lactation yields of Borana cattle in the Southern Rangelands of Ethiopia; 1983. 6.
