# Supplementary material for: Using mixed methods to construct and analyze a participatory agent-based model of a complex Zimbabwean agro-pastoral system
Source: PLoS One. 2020 Aug 21;15(8):e0237638. doi: 10.1371/journal.pone.0237638 (PMC7442250; doi:10.1371/journal.pone.0237638)
Supplement: S3 Appendix — Parameter values, degrees of freedom, test statistics, and p-values from generalized additive statistical model for sensitivity testing of agent-based model results; and additional results figures. (PDF) [file pone.0237638.s003.pdf]

S3 Appendix: Full tables of sensitivity analysis and additional figures. Parameter values, degrees of freedom, test statistics, and p-values from generalized additive statistical model for sensitivity testing of agent-based model results; and additional results figures.

Full GAM results tables

Note: parameters are sorted in terms of decreasing estimated effect size, and all parameters significant at the p<0.05 level are highlighted in bold text.

Table S3.1: Rainfall Scenario parameter estimates for the sustainability model (Chi-squared statistic = 41421, df = 5, p<<0.01). Using the "false discovery rate" correction of Benjamini and Hochberg (1995) gives an adjusted p-value also < 0.001.

Rainfall Scenario	Estimated Effect Size	Transformed Estimate
**random**	**-4.65**	**0.18%**
**statistical-random**	**-4.86**	**0.15%**
**extreme**	**-4.95**	**0.13%**
**historical**	**-5.01**	**0.12%**
**statistical-extreme**	**-6.53**	**0.03%**

Table S3.2: Management Intervention parameter estimates for the sustainability model. See main text for the functional forms for proportion crops and Moran's I. Using the "false discovery rate" correction of Benjamini and Hochberg (1995) gives adjusted p-values also < 0.001.

Management Intervention	Estimated Effect Size	Transformed Estimate	Degrees of Freedom	Chi-squared Statistic	p-value
**s(proportion.crops)**	**smooth function**	**0.42-85.6%**	**8.964**†	**51070**	**< 0.001**
**s(morans.i)**	**smooth function**	**10.2-78.5%**	**8.934**‡	**26008**	**< 0.001**
**how.long.to.store.grain3**	**4.49**	**94.37%**	**1**	**91884**	**< 0.001**
**subsidy.proportion (all categories/levels)**			**4**	**19115**	**< 0.001**
**subsidy.proportiontransport-0.7**	**2.11**	**60.75%**			
**subsidy.proportiontransport-1**	**1.86**	**54.74%**			
**times.per.day.farmers.move.cows1**	**1.13**	**36.70%**	**1**	**12308**	**< 0.001**
**subsidy.proportionfeed-0.7**	**1.12**	**36.46%**			
**key.resources10**	**0.888**	**31.32%**	**1**	**7896.3**	**< 0.001**
**subsidy.proportionfeed-1**	**0.872**	**30.97%**			
**muonde.projects10**	**-0.109**	**14.40%**	**1**	**124.65**	**< 0.001**
**invincible.fencestrue**	**-0.248**	**12.77%**	**1**	**647.51**	**< 0.001**

†df is 'effective df', reference df 9.000
‡df is 'effective df', reference df 8.999

Table S3.3: Underlying variable parameter estimates for sustainability model. P-values corrected for multiple comparisons using the “false discovery rate” method of Benjamini and Hochberg (1995) are shown in the right column.

Underlying variable	Estimated Effect Size	Transformed Estimate	Degrees of Freedom	Chi-squared Statistic	p-value	Adjusted p-value
**woodland.growth.slope**	**0.147**	**17.84%**	**1**	**906.10**	**< 0.001**	**< 0.001**
**catabolism.efficiency**	**0.051**	**16.48%**	**1**	**108.47**	**< 0.001**	**< 0.001**
**kcal.per.kg.of.browse**	**0.045**	**16.40%**	**1**	**86.378**	**< 0.001**	**< 0.001**
**livestock.not.reproduction.rate.per.year**	**0.029**	**16.19%**	**1**	**36.329**	**< 0.001**	**< 0.001**
**kcal.per.kg.of.cow**	**0.014**	**15.99%**	**1**	**8.8572**	**0.003**	**0.004**
kcal.per.kg.of.crop	-0.001	15.79%	1	0.01201	0.913	0.913
production.efficiency	-0.002	15.77%	1	0.16068	0.688	0.708
termite.activity	-0.003	15.76%	1	0.34404	0.558	0.591
wood.to.build.fence.per.meter	-0.003	15.75%	1	0.44784	0.503	0.549
hours.to.plough.ha	-0.006	15.72%	1	1.3256	0.250	0.281
muonde.efficiency	-0.006	15.71%	1	1.6113	0.204	0.237
cow.working.energy.per.hour	-0.007	15.70%	1	2.0833	0.149	0.178
zero.crop.growth.intercept	-0.008	15.69%	1	2.5462	0.110	0.137
calf.birth.mass	-0.010	15.67%	1	3.8277	0.050	0.065
**total.mud.crop.perimeter**	**-0.011**	**15.65%**	**1**	**5.1080**	**0.024**	**0.032**
**crop.growth.slope**	**-0.011**	**15.65%**	**1**	**5.2040**	**0.023**	**0.031**
**max.cow.mass**	**-0.017**	**15.57%**	**1**	**12.042**	**0.001**	**0.001**
**min.cow.mass**	**-0.023**	**15.49%**	**1**	**22.792**	**< 0.001**	**< 0.001**
**cow.maintenance.energy.rate**	**-0.061**	**14.99%**	**1**	**159.64**	**< 0.001**	**< 0.001**

Additional behavioral validation calculations and figures

These three metrics are on the same order of magnitude as the field data/calculations, despite not having been used for any form of calibration.

Figure S3.1: Field-based data shown in dark purple and distributions of model summary variables in teal; in these cases, we have only single (or high and low) estimates from data (vertical lines). A) Minimum woodland biomass, with the 2013 estimate of woodland biomass (vertical dark purple line) shown for comparison with the model; B) The amount of crop eaten in kg/hour/cow, with the calculations from data resulting in a high and low estimates (vertical dark purple lines); C) The amount of subsidy in 1000s of USD/year.

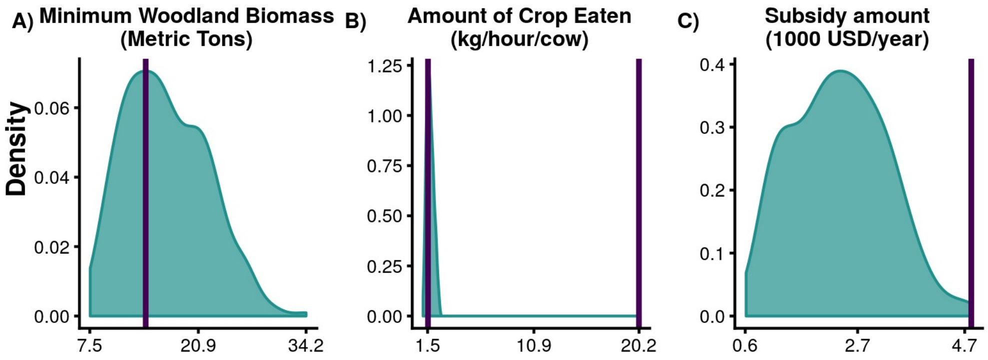

Figures showing spatial variables stratified by proportion crops, and distribution of those variables in those subsets

Figure S3.2: Model sustainability vs spatial variables for ranges of proportion-crops

A) Proportion crops 0-20% B) Proportion crops 35-55% C) Proportion crops 80-100%

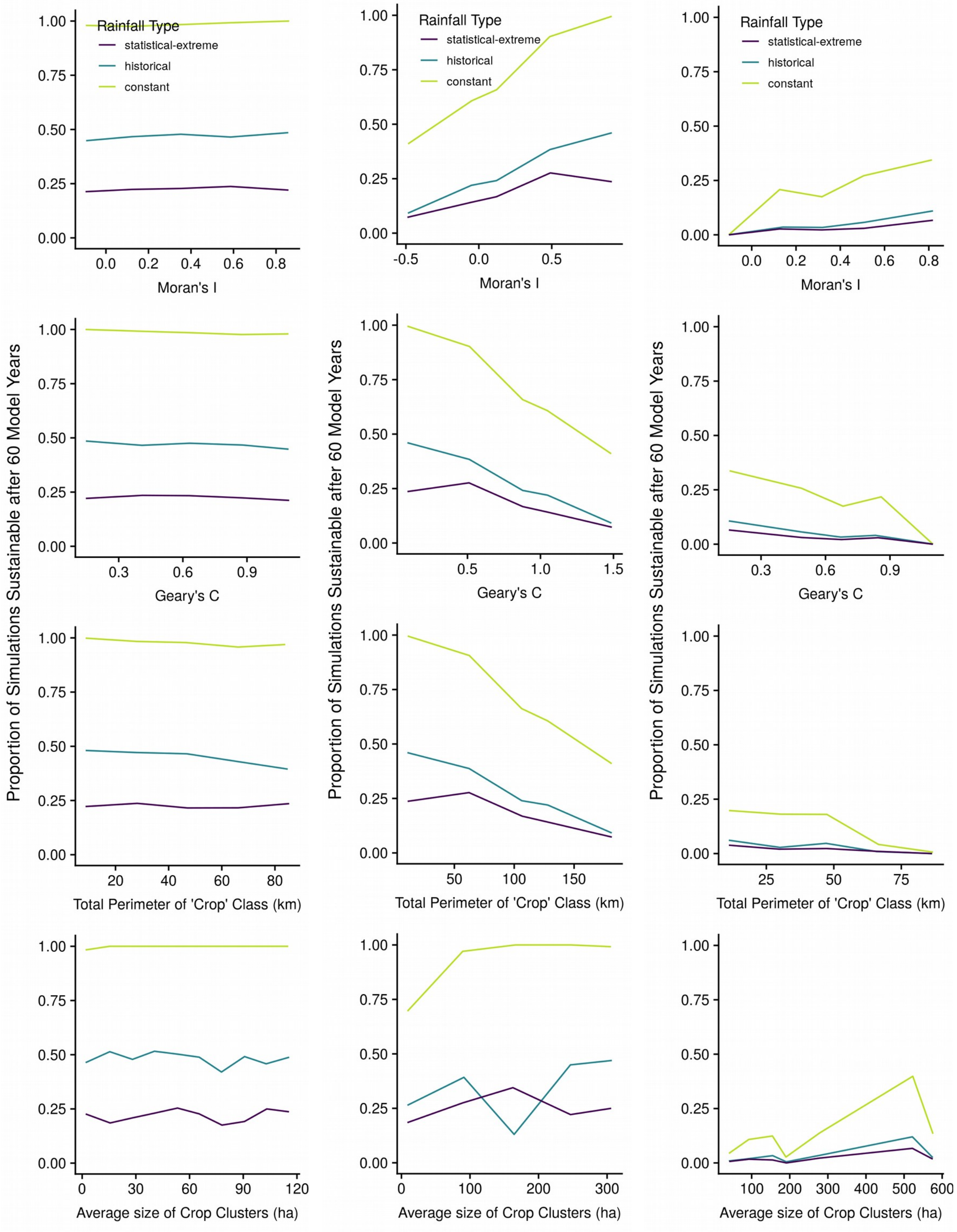

Figure S3.3: Distribution of spatial variables for ranges of proportion-crops

A) Proportion crops 0-20% B) Proportion crops 35-55% C) Proportion crops 80-100%

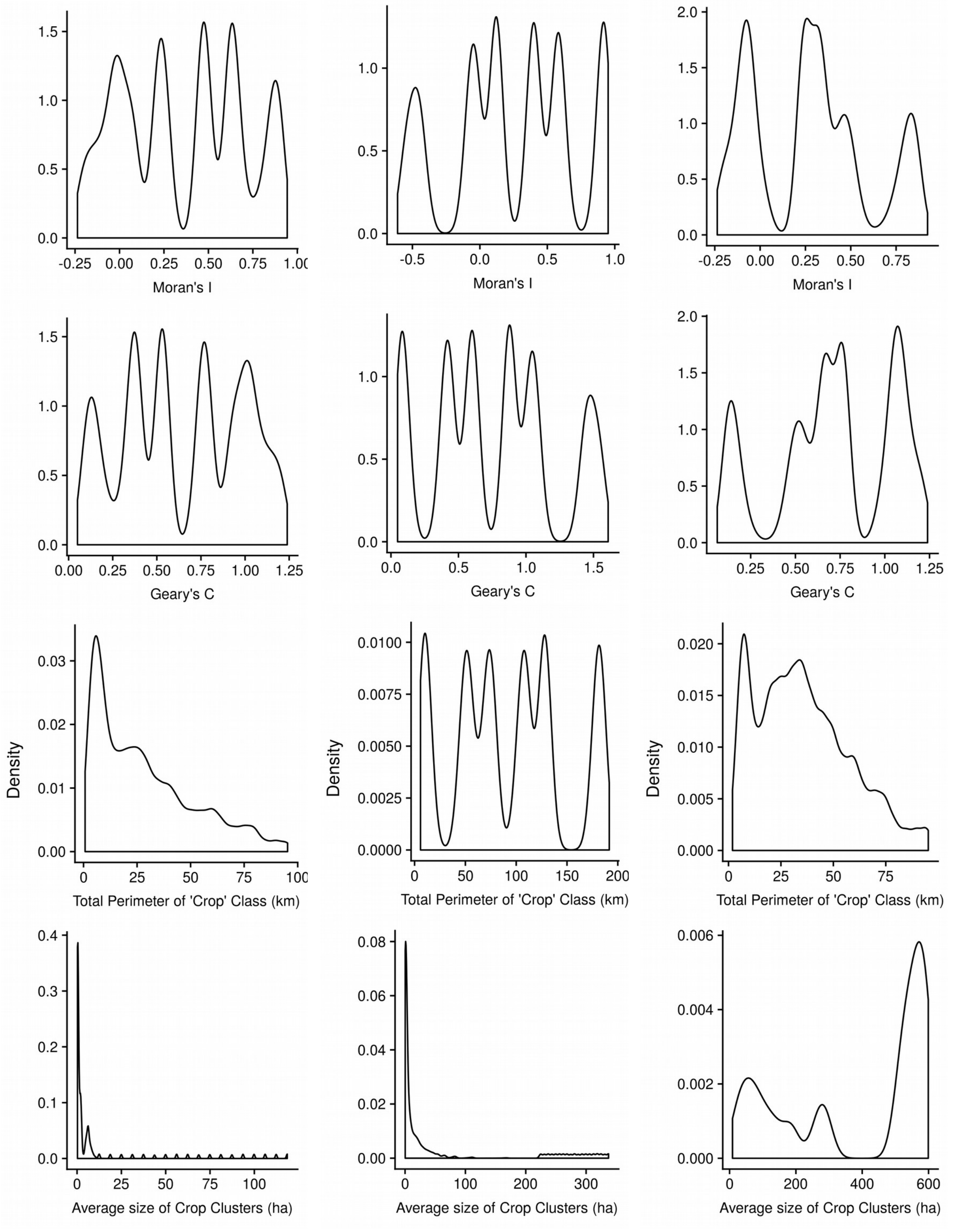

Figure S3.4: Distribution of spatial variables for all proportions of crops (0-100%)

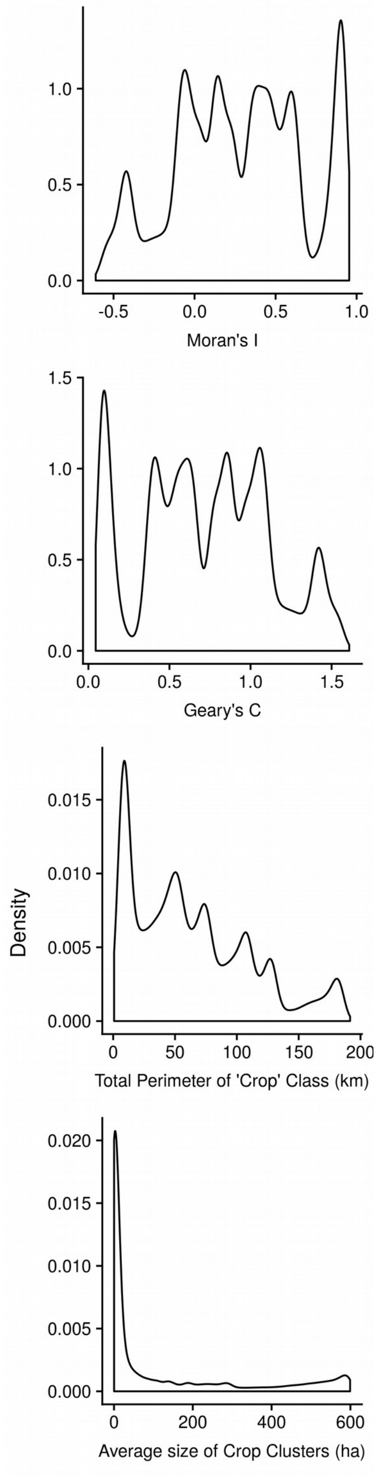

Figures showing all combinations of rainfall scenarios, spatial variables, and management interventions

Figure S3.5: As Fig. 7, but including all the rainfall scenarios (other than 'constant'), and showing possible interactions between interventions and rainfall scenarios.

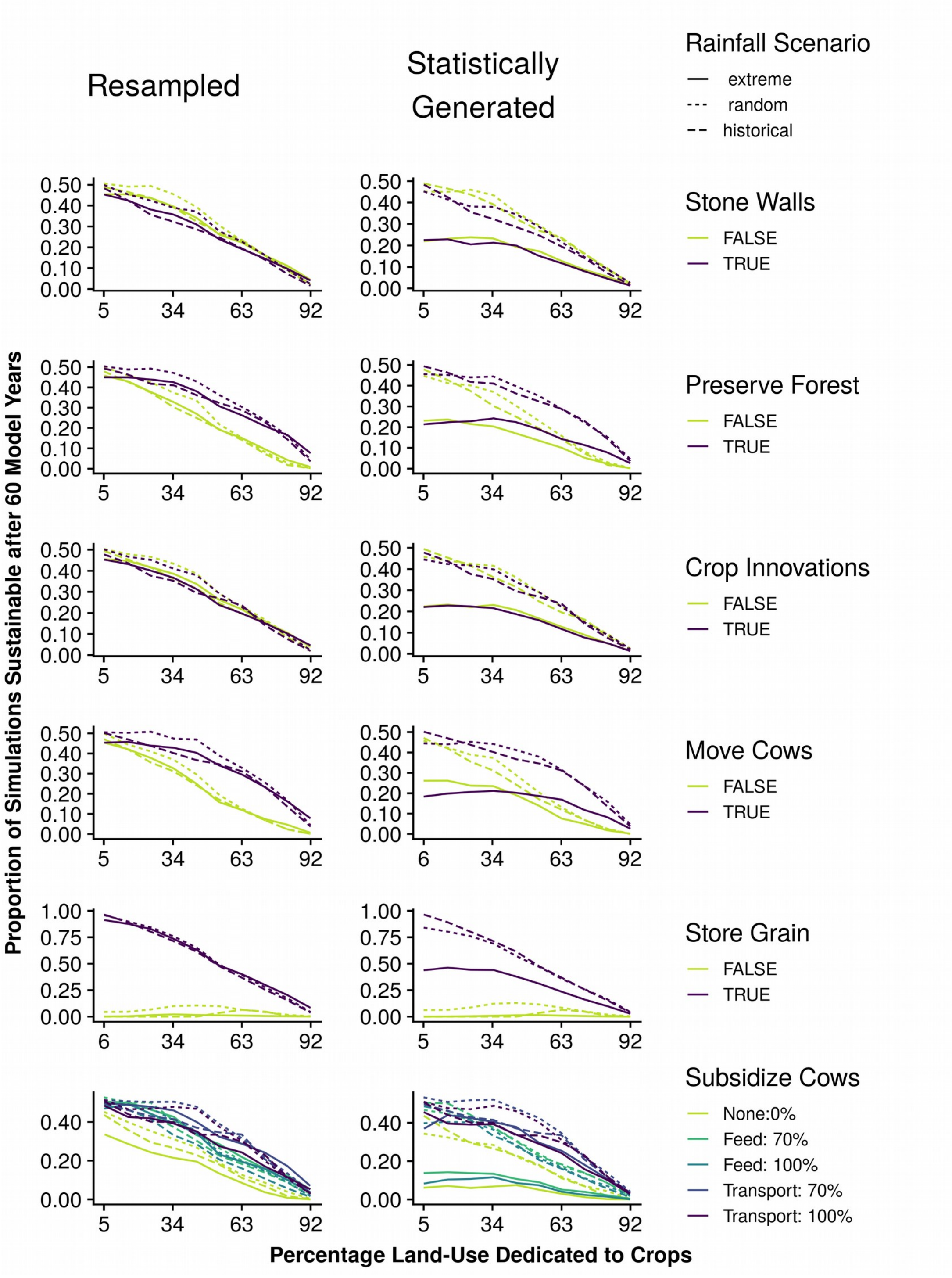

Figure S3.6: As Fig. S3.5, but for Moran's I, and including all the rainfall scenarios (other than 'constant').

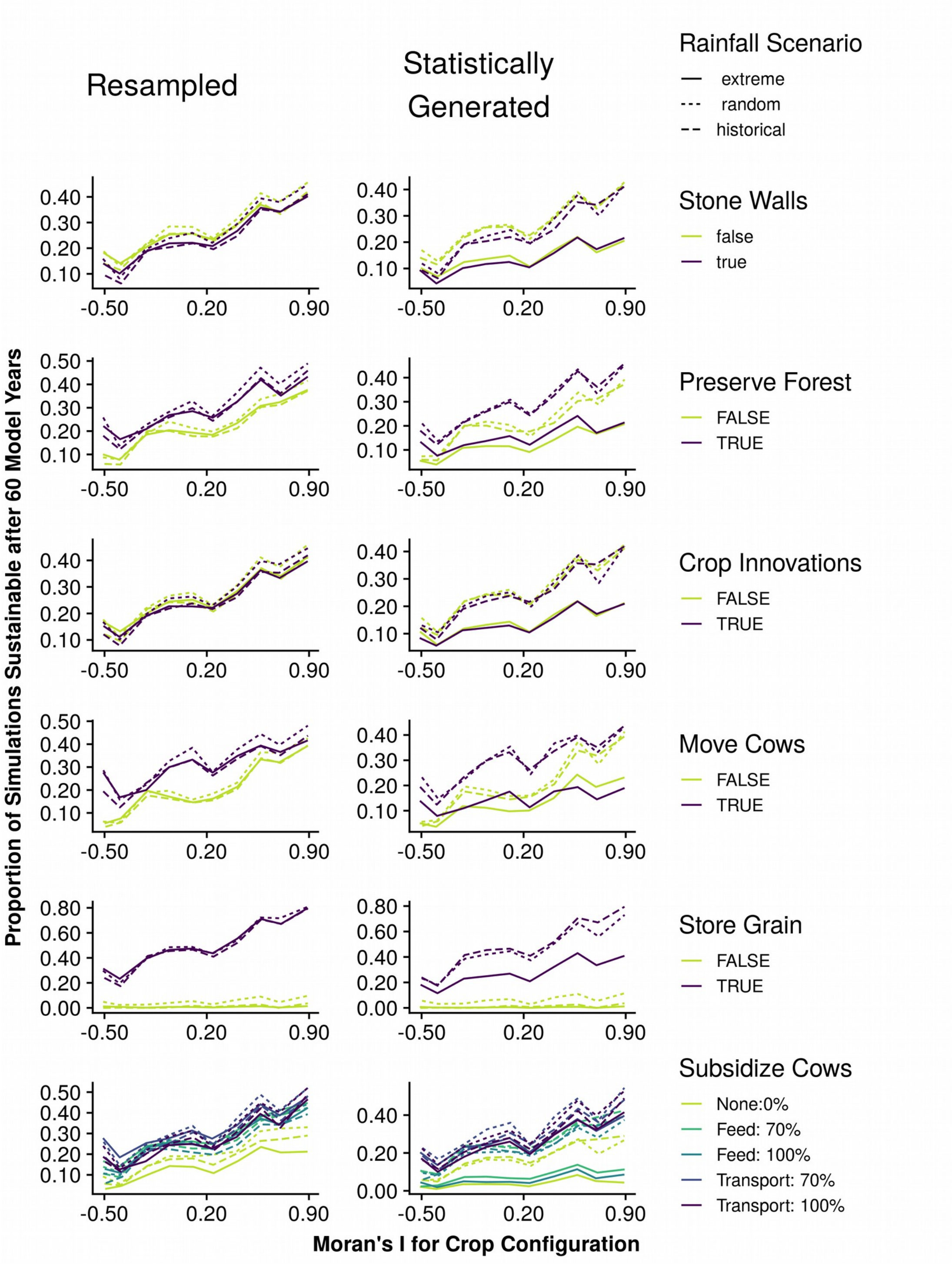

Figure S3.7: As Fig. S3.5, but for Geary's C and including all the rainfall scenarios (other than 'constant').

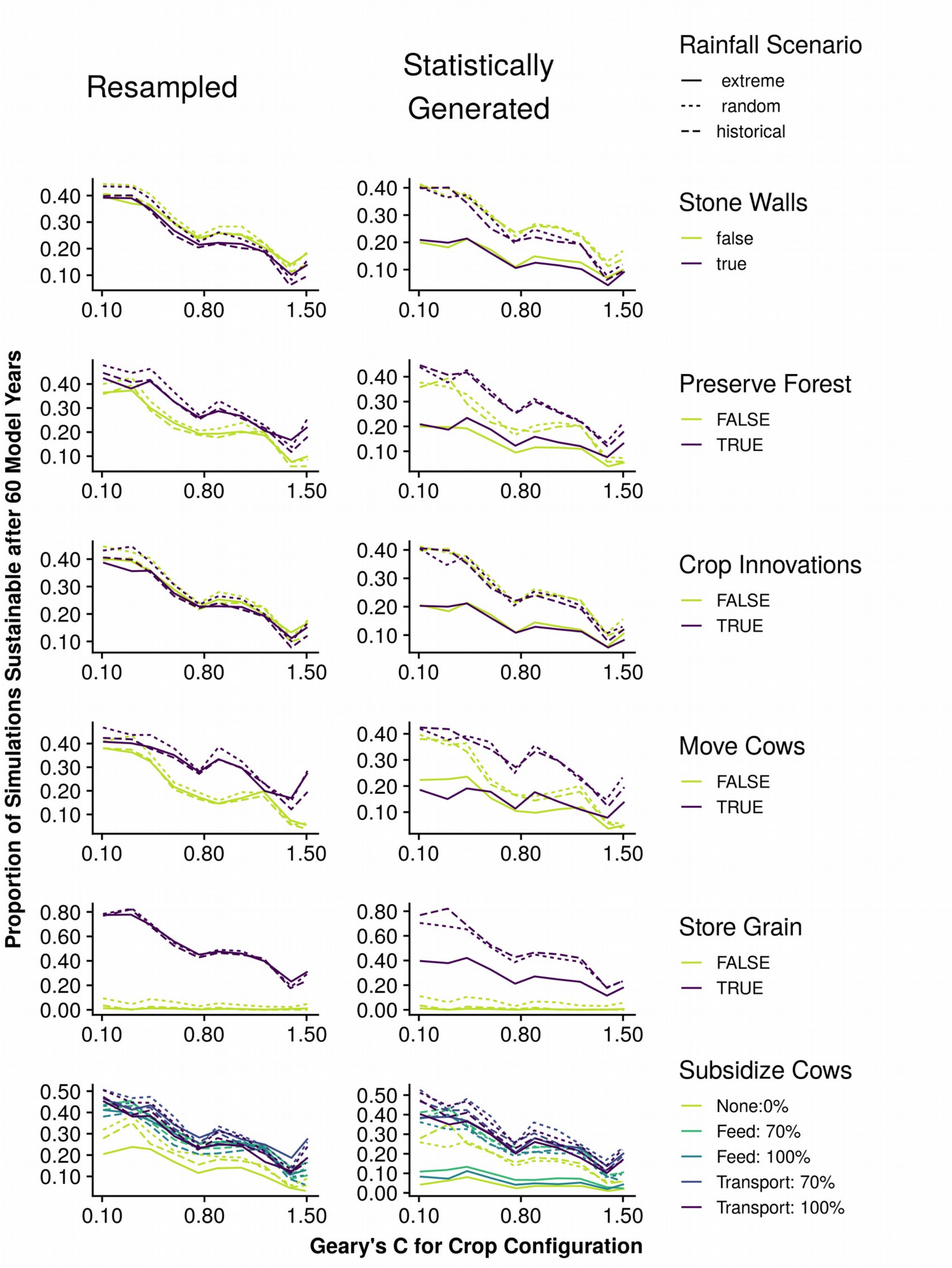

Figure S3.8: As Fig. S3.5, but for the total perimeter of the ‘crop’ class, and including all the rainfall scenarios (other than ‘constant’).

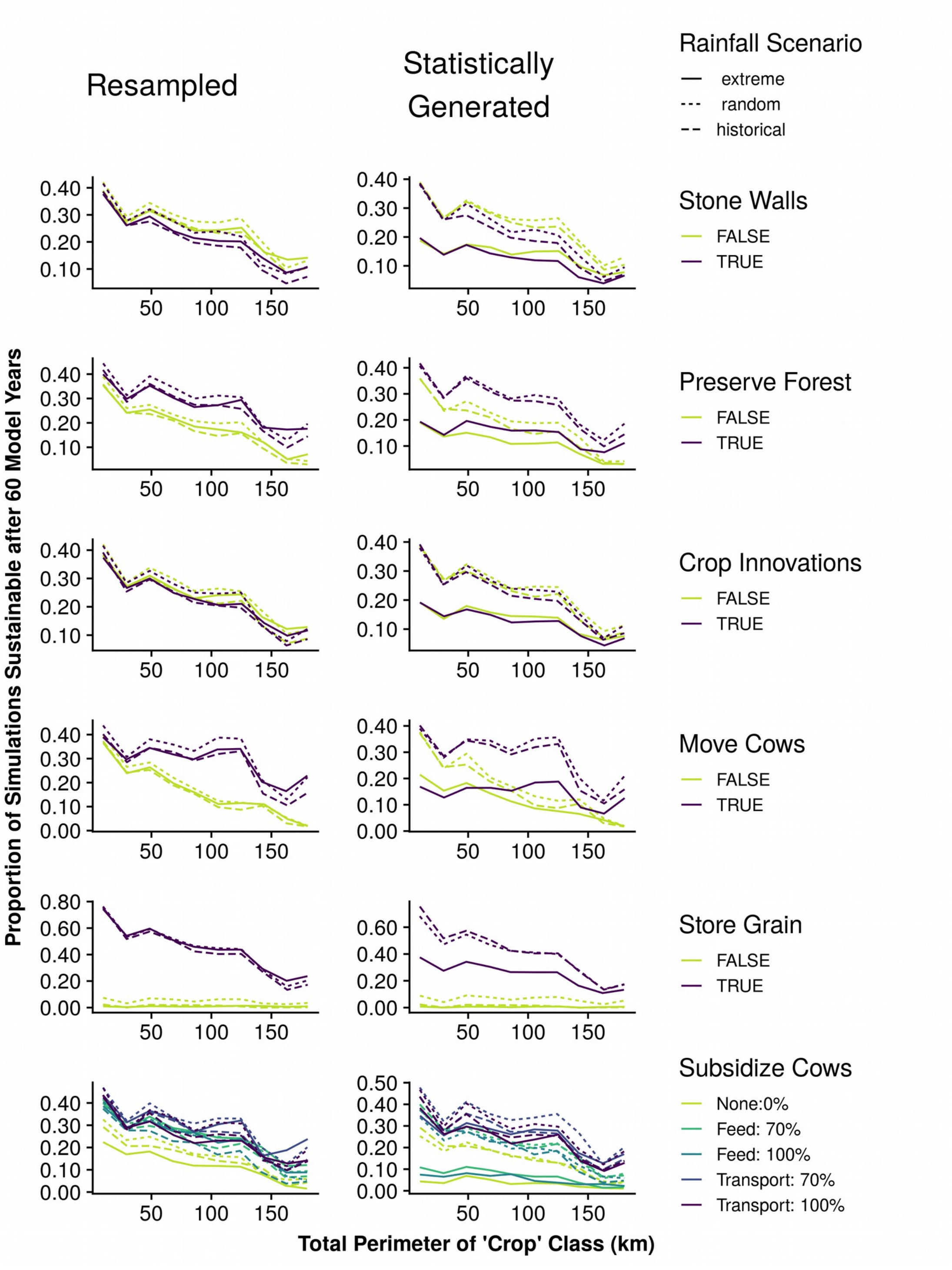

Figure S3.9: As Fig S3.5, but for the average crop cluster size, and including all the rainfall scenarios (other than ‘constant’).

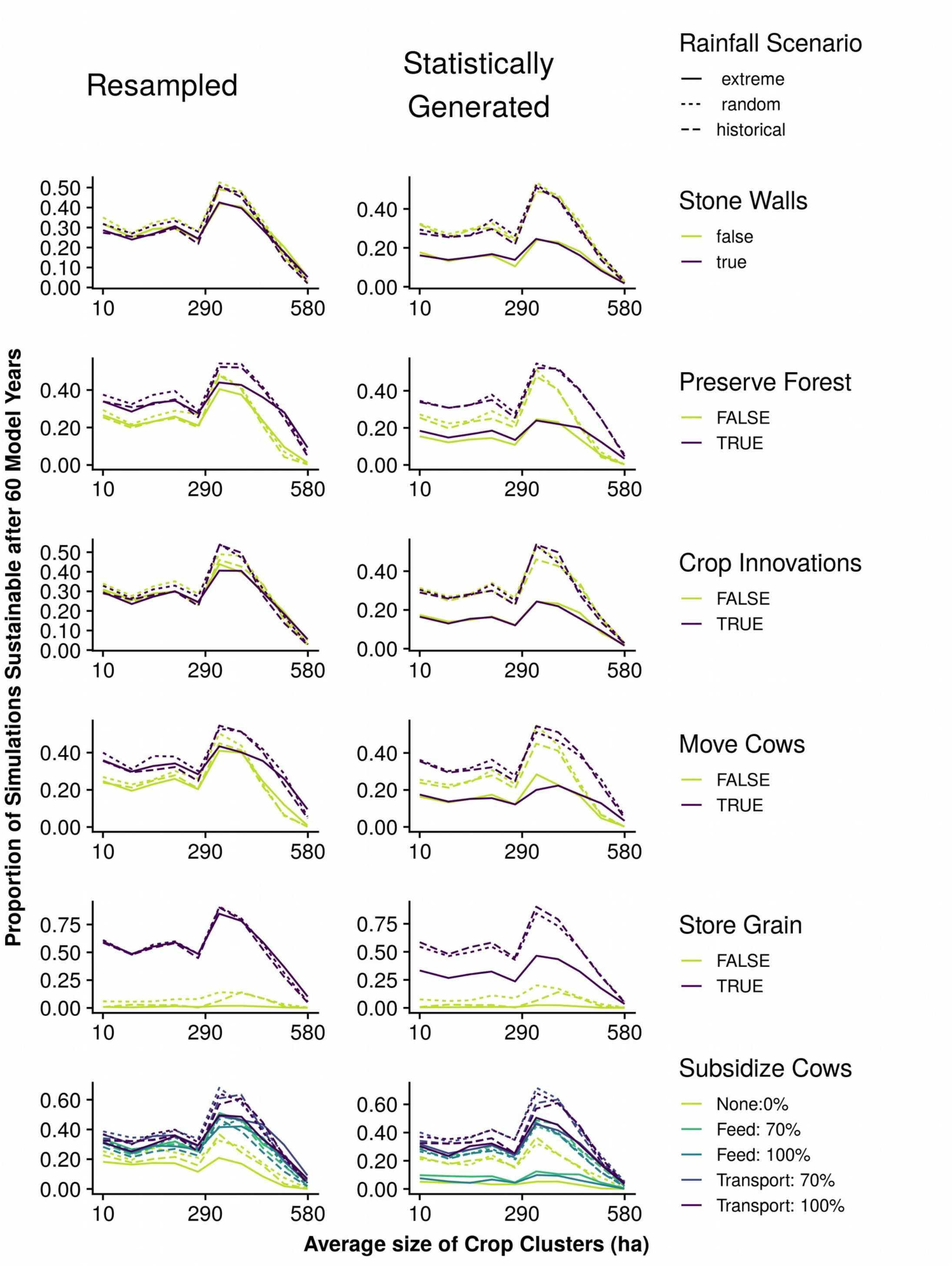

References

Benjamini, Y., and Hochberg, Y. (1995). Controlling the false discovery rate: a practical and powerful approach to multiple testing. Journal of the Royal Statistical Society Series B, 57, 289–300.
